# Characterizing expression changes in noncoding RNAs during aging and heterochronic parabiosis across mouse tissues

**DOI:** 10.1038/s41587-023-01751-6

**Published:** 2023-04-27

**Authors:** Viktoria Wagner, Fabian Kern, Oliver Hahn, Nicholas Schaum, Nicole Ludwig, Tobias Fehlmann, Annika Engel, Dominic Henn, Shusruto Rishik, Alina Isakova, Michelle Tan, Rene Sit, Norma Neff, Martin Hart, Eckart Meese, Steve Quake, Tony Wyss-Coray, Andreas Keller

**Affiliations:** 1 Clinical Bioinformatics, Saarland University, Saarbrücken, Germany; 2 Department of Neurology and Neurological Sciences, Stanford University, Stanford, CA USA; 3 Helmholtz Institute for Pharmaceutical Research Saarland (HIPS)–Helmholtz Centre for Infection Research (HZI), Saarland University Campus, Saarbrücken, Germany; 4 Department of Human Genetics, Saarland University, Saarland, Germany; 5 Department of Plastic Surgery, University of Texas Southwestern Medical Center, Dallas, TX USA; 6 Department of Bioengineering, Stanford University, Stanford, CA USA; 7 The Phil and Penny Knight Initiative for Brain Resilience, Stanford University, Stanford, CA USA

**Keywords:** miRNAs, Computational biology and bioinformatics, Diagnostic markers, Geriatrics, Ageing

## Abstract

Molecular mechanisms of organismal and cell aging remain incompletely understood. We, therefore, generated a body-wide map of noncoding RNA (ncRNA) expression in aging (16 organs at ten timepoints from 1 to 27 months) and rejuvenated mice. We found molecular aging trajectories are largely tissue-specific except for eight broadly deregulated microRNAs (miRNAs). Their individual abundance mirrors their presence in circulating plasma and extracellular vesicles (EVs) whereas tissue-specific ncRNAs were less present. For miR-29c-3p, we observe the largest correlation with aging in solid organs, plasma and EVs. In mice rejuvenated by heterochronic parabiosis, miR-29c-3p was the most prominent miRNA restored to similar levels found in young liver. miR-29c-3p targets the extracellular matrix and secretion pathways, known to be implicated in aging. We provide a map of organism-wide expression of ncRNAs with aging and rejuvenation and identify a set of broadly deregulated miRNAs, which may function as systemic regulators of aging via plasma and EVs.

## Abstract (web summary)

An atlas of noncoding RNA in aging and rejuvenated mice identifies a set of deregulated miRNAs.

## Main

One primary risk factor for cancer, diabetes, cardiovascular disorders and neurodegenerative diseases is aging^1^. Therefore, understanding the underlying mechanisms of this complex process is essential to improve quality of life by developing new therapies. Finding the most promising therapy target is challenging, as it is not possible for a single level of omics data to explain whether the changes discovered are causative to or the result of aging^2^. Epigenetic markers like DNA methylations have been identified as promising aging biomarkers^3,4^. Current research efforts, including transcriptomic studies of major organs in aged mice^5,6^, largely lack information covering the whole RNA diversity, for example, the diverse classes of noncoding RNAs (ncRNAs). Attempting to better differentiate cause and effect, we herein present the corresponding ncRNA dataset to the Tabula Muris Senis (TMS) cohort^5^. Such RNAs are part of epigenetic reprogramming and altered intercellular communication, which have been described as hallmarks of aging^1,7^. Further, they can have a role in intercellular communication via extracellular vesicles (EV)^8^. MicroRNAs (miRNA), a class of ncRNAs, target messenger RNA (mRNA) through base-pair binding and thereby regulate gene expression via post-transcriptional gene silencing^7,9^. Furthermore, miRNAs act as age-specific disease biomarkers^10^ and have been identified as regulators in aging-associated phenotypes^11^.

We analyzed eight classes of ncRNAs in TMS separately and together with the existing single-cell and bulk mRNA datasets^5^. The previously observed tissue-driven shifts in gene expression with aging that correlate with corresponding protein levels in plasma could be caused by epigenetic regulation mechanisms mediated by ncRNA. Furthermore, these may not only be implicated in aging but also have a role in the regenerative effects observed in aging interventions such as heterochronic parabiosis. Regenerative activities within young blood with translational implications for aged liver, muscle and brain have been observed before^12^. Therefore, we performed ncRNA sequencing of tissue samples following heterochronic parabiosis experiments, in which a young (3–4 months) and an aged (19 months) mouse share a common blood circulation. Our two datasets describe age- and rejuvenation-related ncRNA expression changes to reveal the potential of ncRNAs as targets for new pharmaceutical approaches.

## Results

### Mapping of ncRNA expression across mouse organs

**Fig. 1:**
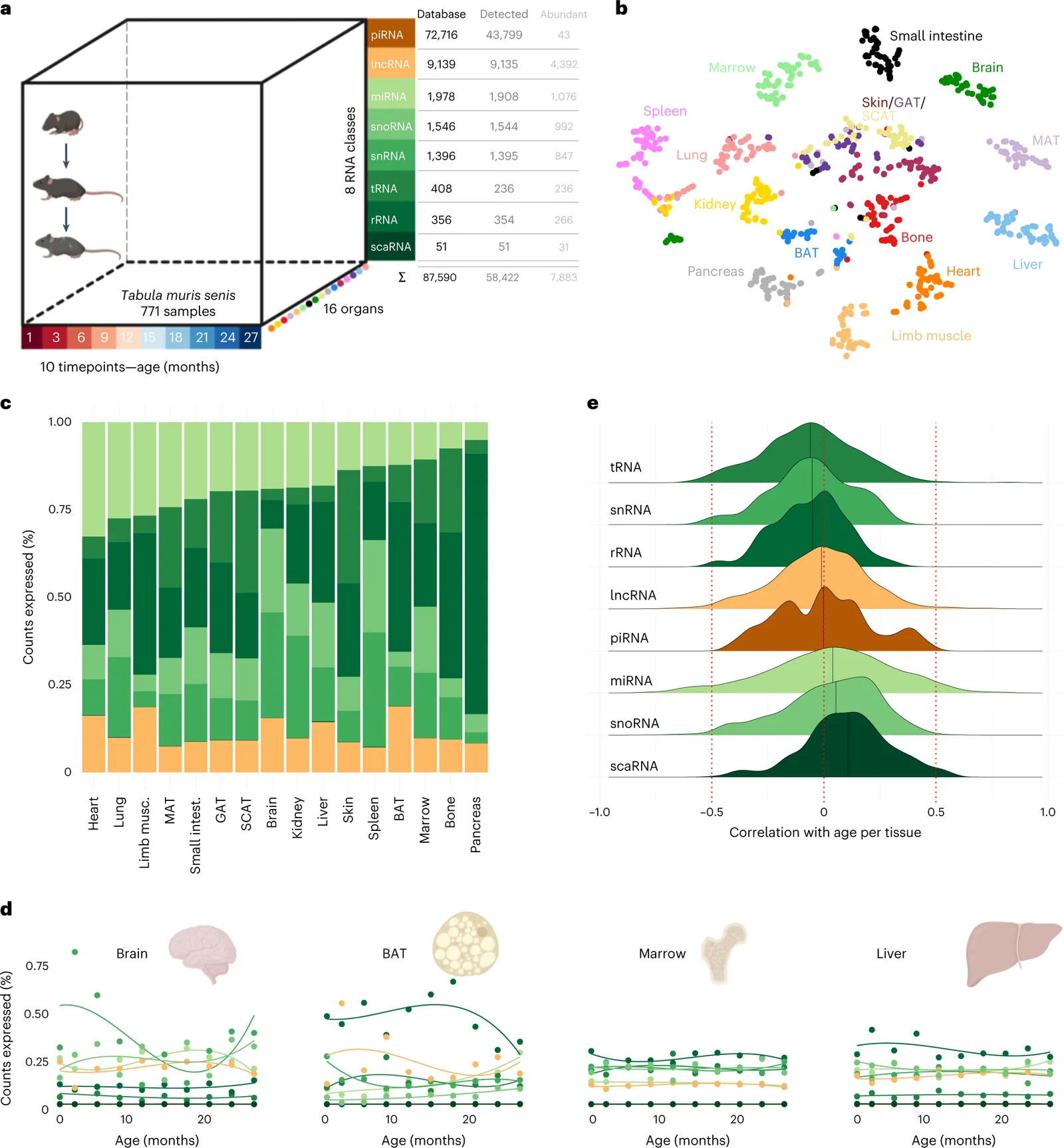
Atlas of noncoding RNA expression along the mouse lifespan. **a**, Study overview—data of the aging (TMS) cohort, consisting of mouse samples collected from 16 different tissues at ten different timepoints throughout the lifespan with maximal six replicates per timepoint varying due to sample and sequencing quality. A total of 771 samples were sequenced, and the reads were annotated to the 87,590 different RNA reference sequences from eight RNA classes. Of the RNAs in the databases listed on the left, 58,422 different ncRNAs were annotated in the raw reads and we found 7,883 noncoding features as abundant expressed in our TMS aging cohort. Created with BioRender. **b**, t-SNE visualization of all samples of the TMS cohort over all detected noncoding RNAs, colored by tissue of origin. **c**, Percentage of counts per RNA class, calculated on total counts per tissue after local filtering for all tissues in the TMS cohort, color coded by RNA-class color legend as indicated in **a**. **d**, Variation of mean count distribution per RNA class over the lifespan of the mouse in the brain, BAT, marrow and liver; calculated count percentages per sample after local filtering. Created with BioRender. **e**, Density plot of Spearman rank correlation of all expressed noncoding RNA with age in each individual tissue grouped by RNA classes, density scaled individually for every RNA class.

We sequenced 771 tissue samples of the TMS cohort to map molecular shifts across the whole organism during healthy aging (Fig. 1a). The protocol enriches for small ncRNA, especially mature miRNAs. Even though full-length reads cover only small ncRNAs (miRNAs or piwi-interacting RNAs (piRNAs)) completely, the protocol generates measurable fragments of longer ncRNAs. This sequencing strategy extends the existing mRNA TMS dataset^5^ with miRNA, piRNA, long ncRNA (lncRNA), small nucleolar RNA (snoRNA), small nuclear RNA (snRNA), transfer RNA (tRNA), ribosomal RNA (rRNA) and small Cajal body-specific RNA (scaRNA). The tissue sample collection includes 16 solid tissues of C57BL6/JN mice (bone (femurs and tibiae), brain (hemibrain), brown adipose tissue (BAT, interscapular depot), gonadal adipose tissue (GAT, inguinal depot), heart, kidney, limb muscle (tibialis anterior), liver, lung, bone marrow, mesenteric adipose tissue (MAT), pancreas, skin, small intestine (duodenum), spleen and subcutaneous adipose tissue (SCAT, posterior depot)). The selected time course covers the mouse lifespan from a developmental age of 1 month up to 27 months (males: aged 1, 3, 6, 9, 12, 15, 18, 21, 24 and 27 months; females: aged 1, 3, 6, 9, 12, 15, 18 and 21 months). With up to six mice per timepoint, the study covers a maximum of 960 samples (16 organs × 10 timepoints × 6 replicates). As not all mice survived to the later timepoints and we further excluded 26 low-quality RNA samples, we finally included 771 high-quality samples in the study (Supplementary Table 1).

**Extended Data Fig. 1:**
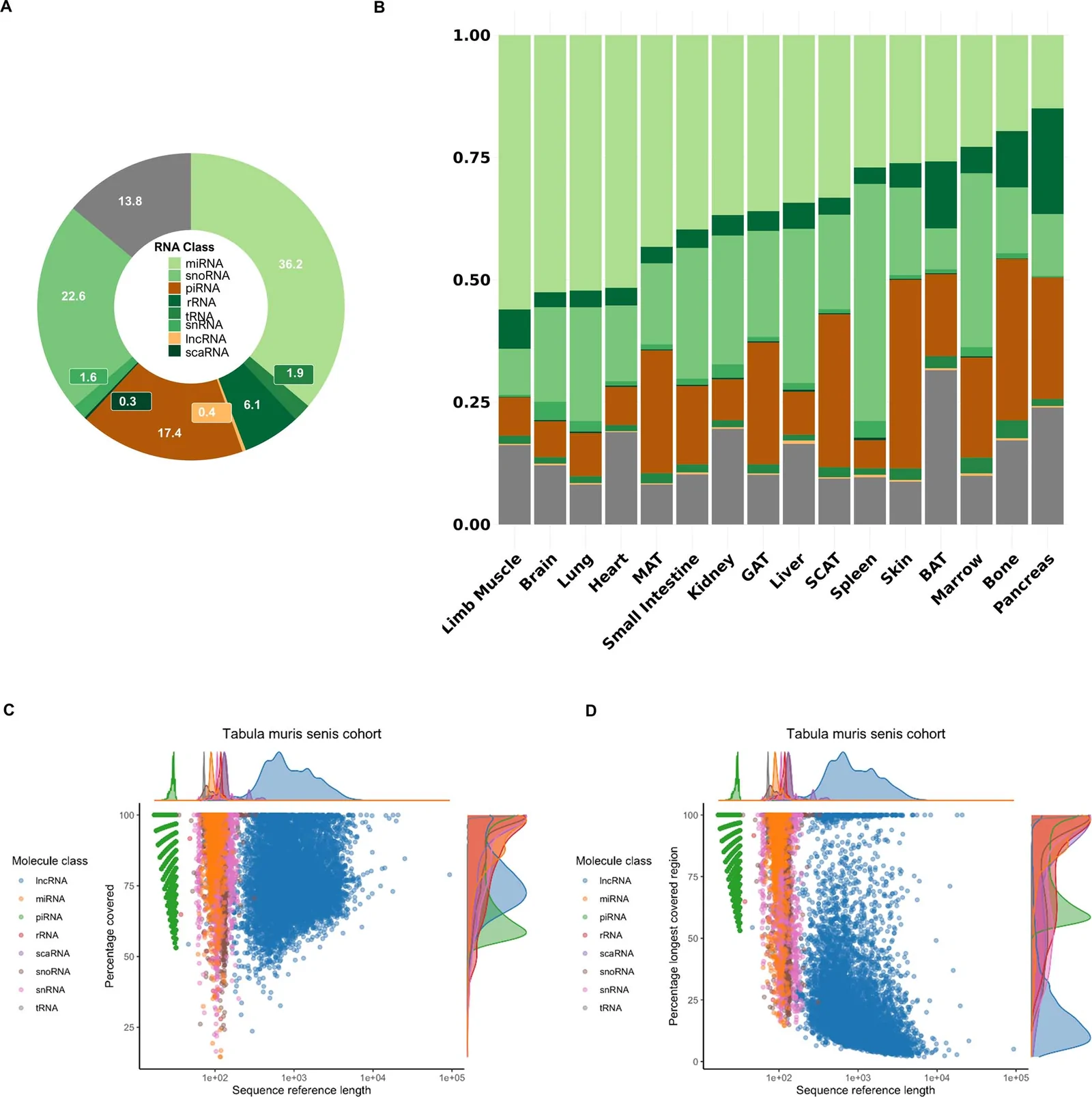
Non-coding RNA read distribution. (A) Averaged percentage of mapped reads for all RNA classes over all TMS cohort samples. (B) Mapped RNA class read distribution in percent for each tissue, sorted descending by miRNA share. (C) Covered length analysis: Percentage covered of reference sequence with mapped reads versus sequence reference length, colored by RNA class for all ncRNAs detected in the raw reads for the TMS cohort (D) Percentage of longest covered region calculated with the maximal connected read length versus sequence reference length for all ncRNAs detected in the raw reads for the TMS cohort.

We mapped resulting sequencing reads against 87,590 ncRNA sequences (Fig. 1a, left column) derived from established reference databases (miRNAs, miRBase 22, tRNAs: GtRNAdb 18.1, piRNA: RNACentral 15, all other ncRNAs: Ensembl 100). Altogether, we detected reads mapping to 58,422 different ncRNAs (Fig. 1a, middle column), with miRNAs being the most abundant class. An average of 36.2% of reads across tissues mapped to miRNAs (Extended Data Fig. 1a). The distribution of reads to RNA classes, however, varied substantially between tissues (*P* < 0.05, Kruskal–Wallis test; Extended Data Fig. 1b). We thus asked whether the variation in read distribution is related to the length of representatives. We generated aligned sequence profiles to quantify the length of sequences covered with our reads. First, we explored the percentage of sequence length covered versus the sequence reference length. Even for very long sequences exceeding 10,000 bases, we partially recovered large fractions or even the complete sequence (Extended Data Fig. 1c). We also computed the maximal assembly for each RNA, that is, the longest contiguous read mapping, and compared it with the sequence reference length (Extended Data Fig. 1d). Even though the fraction of the sequence covered by the maximal assembly decreased for larger RNAs (Spearman’s rho = −0.43), we verified that throughout all RNA classes, we were still able to reproduce for a subset up to 100% of the full-length reference (Supplementary Table 2). Therefore, we decided to include the full dataset as a reference for future studies while implementing restrictive filtering steps to increase the reliability of our data. Especially the somatic piRNAs exceeded the expected counts, likely driven by artifacts in piRNA annotation^13^ and calling for additional quality control filters. We first retained piRNAs encoded in prepachytene piRNA genomic clusters^14,15^ to minimize the number of false positive hits. Next, we removed low abundant features across all ncRNA classes, keeping those with at least 1 read mapped per million (rpmm) in at least one sample, resulting in the abundant dataset (Fig. 1a, right column). Applying this stringent filtering, the number of piRNAs in our dataset decreased from 43,799 detected down to 43 abundant, likely removing most falsely annotated features^13^.

**Extended Data Fig. 2:**
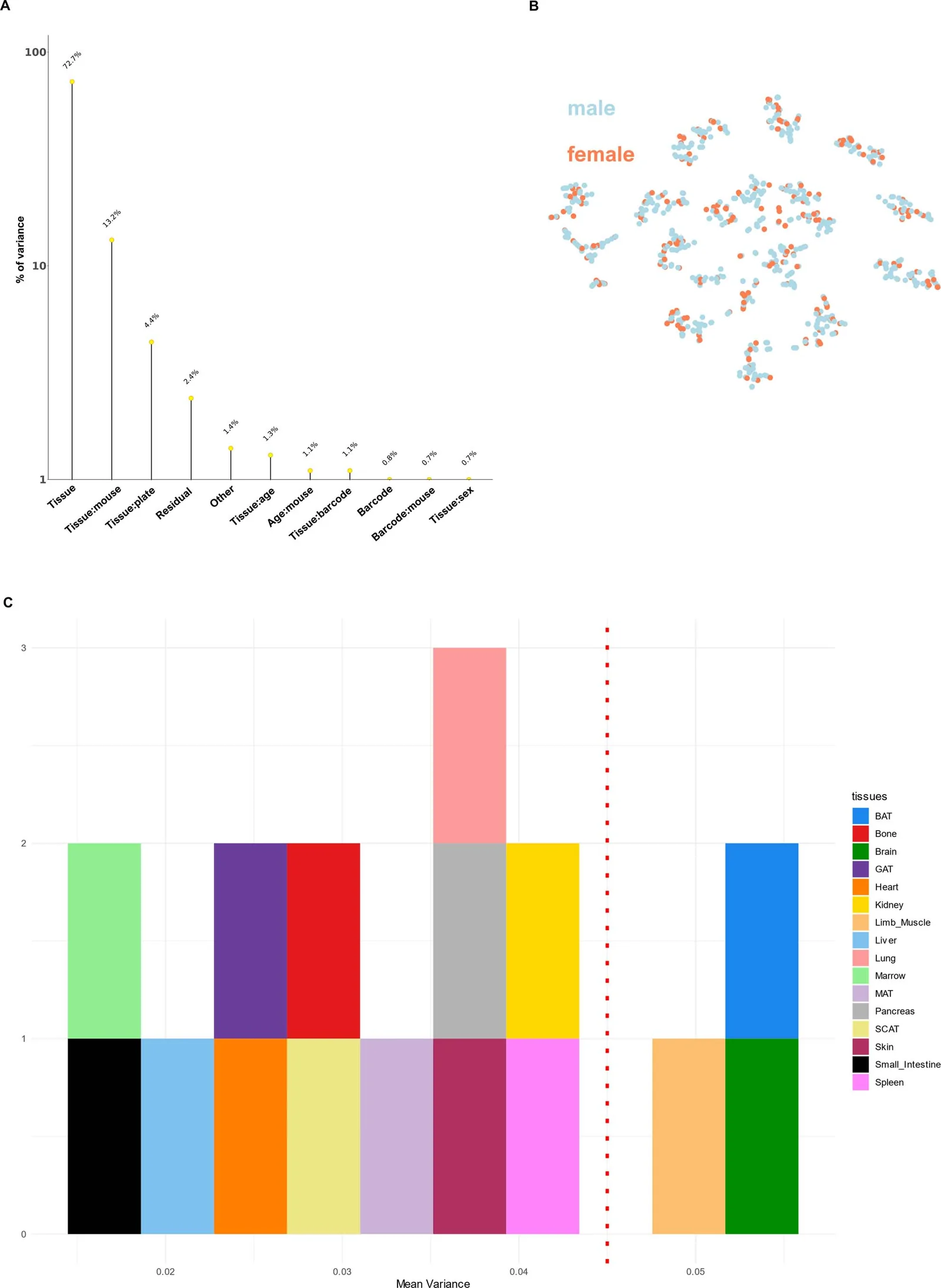
Variance analysis for ncRNA expression levels. (A) Principal variance component analysis of biological components (sex, age, tissue, mouse) and technical components (barcode, plate); factors connected with ':' were linearly combined. (B) Visualization of all samples of the TMS cohort over all abundant non-coding RNAs as a t-SNE, colored by sex. (C) Histogram of mean variance calculated in abundant count percentages of all RNA classes. Calculations for each tissue after local filtering over the time course. Threshold determined at 4.5% to separate highly variable and stable tissues.

Clustering by ncRNA expression using t-distributed stochastic neighbor embedding (t-SNE), samples split into tissue-specific groups (Fig. 1b). One cluster contained skin, GAT and SCAT samples, which likely can be explained by their biological and functional close relationship of containing similar cell types. To check whether relevant biological factors outweigh technical ones, we performed a principal variance component analysis. The highest proportion of variance in the data was explained by tissue identity (Extended Data Fig. 2a). Annotating the t-SNE plot by animal sex revealed a uniform spread, excluding it as a major driver of the observed variance (Extended Data Fig. 2b).

**Extended Data Fig. 3:**
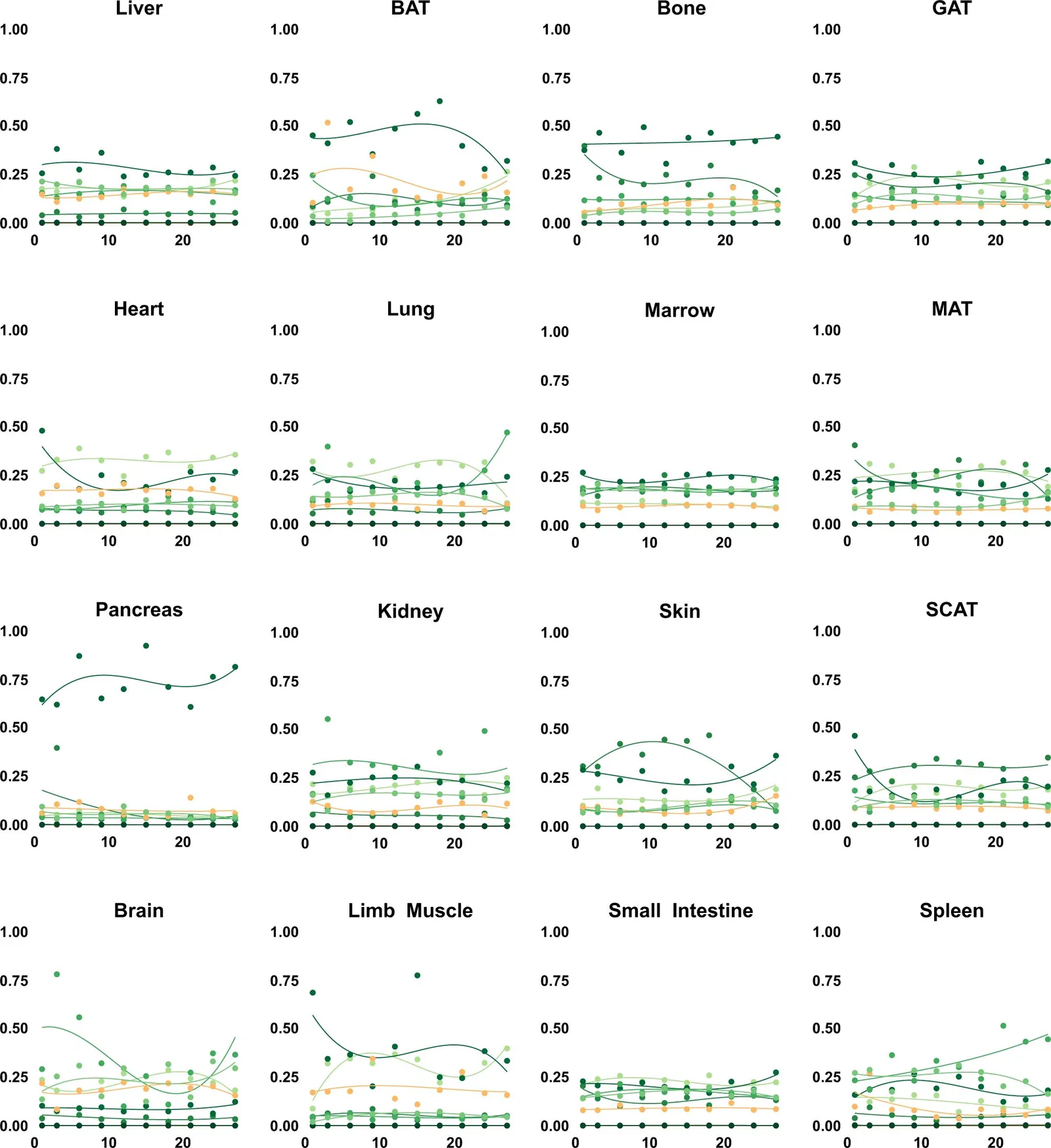
Mean count percentages per RNA class over the lifespan in all organs. Mean count percentages per RNA class and over all timepoints, for each tissue in the TMS dataset. Calculations based on count shares per sample after local filtering.

Following our main objective to identify organ-specific aging trajectories, we added a tissue-specific, that is, local filtering to check whether ncRNA expression changed not only between tissues but also with age (cf. Methods). On the locally-filtered data, we calculated read count percentages for all RNA classes. As for the detected reads, we observed tissue-specific distributions (Fig. 1c). Analyzing those over time, we identified two clusters of tissues (Extended Data Fig. 2c). One exhibited a stable count distribution (mean variance < 4.5%) and the other showed high variance within the count distribution (mean variance > 4.5%). Specifically, 3 of 16 tissues showed high variance (brain, BAT and limb muscle), while most tissues (13–16) were characterized by a stable read distribution (including marrow and liver) (Fig. 1d and Extended Data Fig. 3). In the brain, the share of snRNA reads decreased from 77.9% to 10.0%, while the share of miRNAs increased from 9.1% to 28.5%. In BAT, the miRNA share grew steadily from 4.1% to 26.4% and the rRNA share dropped from 62.7% to 27.8%.

The observed variations of the RNA classes prompted us to assess the expression changes during aging for the individual ncRNAs. Therefore, we determined the Spearman rank correlation of age with the expression of every ncRNA in each tissue separately. We identified 31 tRNA fragments that were substantially differentially expressed between 3 and 21 months (two-sided *t*-test, *P* adjust < 0.05). Eight tRNA fragments showed increased expression (in brain and lung) and 23 showed decreased expression with age (in bone, limb muscle, skin and GAT). tRNA-related metabolism, transcription, modification and derivatives have vital roles in aging and longevity of organisms, as tRNA expression decreases with age^16^. We further observed that miRNAs displayed the strongest correlations with age over all tissues (exceeding the interval of −0.5 to 0.5; Fig. 1e). Given that miRNAs were captured in full-length by our sequencing platform, their high abundance across tissues and the fact that they exhibited the largest effect size, we further focused on miRNAs for downstream analysis.

### MiRNA lifespan trajectories are largely tissue specific

**Fig. 2:**
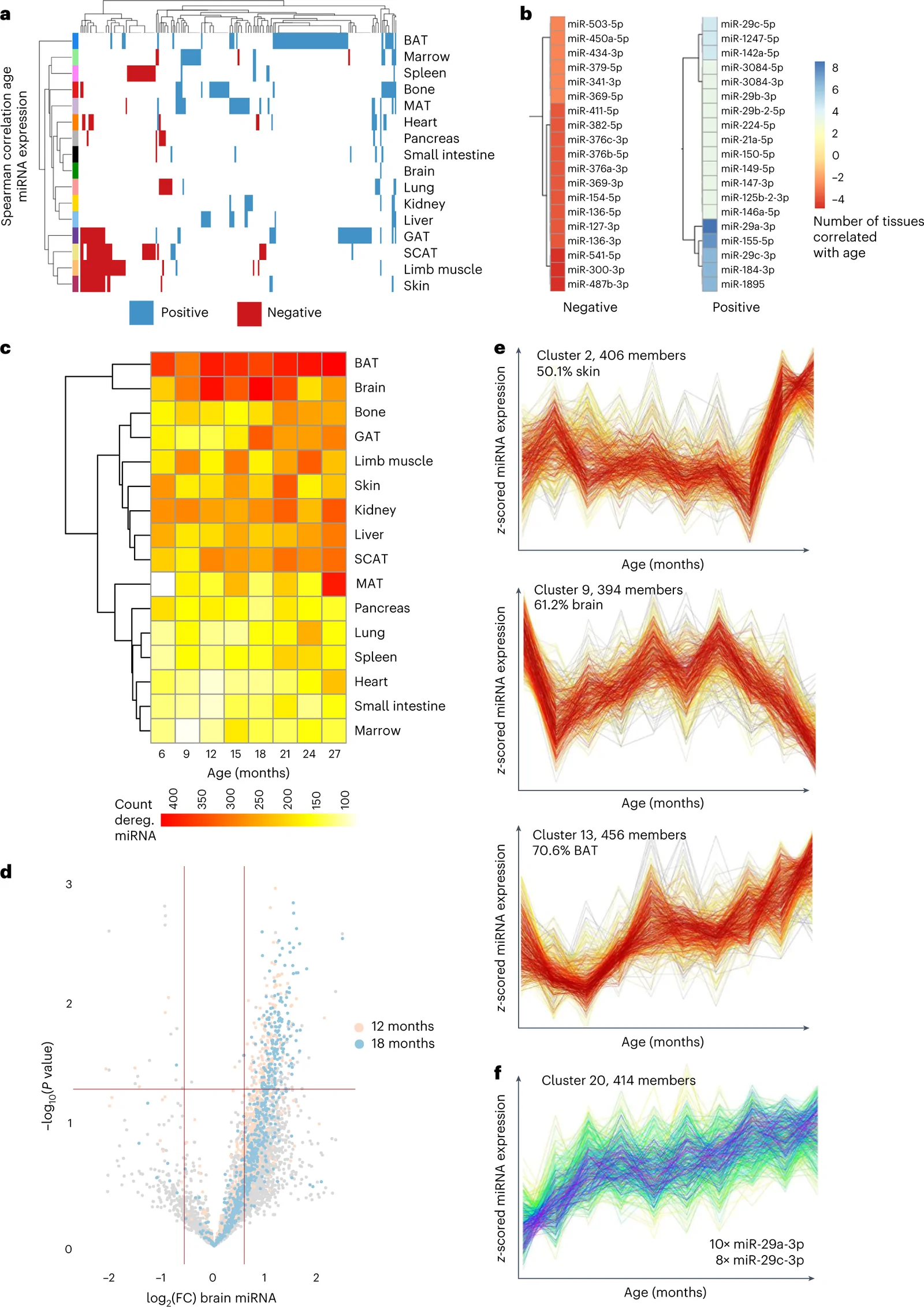
Global and tissue-specific miRNA expression patterns with aging. **a**, Heatmap of Spearman rank correlation values of the intersection of miRNAs expressed in all tissues, color coded for positively correlated in blue (*r* > 0.5), negatively correlated in red (*r* < −0.5) and not correlated in white (−0.5 < *r* < 0.5). **b**, Heatmap of miRNAs (anti-) correlated with age in at least two tissues, colored by number of tissues (anti-) correlated and divided into miRNA positively and negatively correlated with age. **c**, Heatmap for the count of deregulated miRNAs in each tissue at each subsequent timepoint. Deregulated miRNAs are determined by calculating the foldchange of all later timepoints versus 3 months of age and miRNA with foldchanges <2/3 or >3/2 are considered deregulated. **d**, Volcano plot of all miRNAs expressed in brain, log_2_(FC) versus −log_10_(*P* values) (two-sided *t*-test) calculated between mice aged 3 months and all later timepoints with comparisons for 12 months (light red) and 18 months (light blue) highlighted. **e**, Whole organism miRNA trajectory clustering—*z*-scored trajectories of each expressed miRNA in each tissue over the entire lifespan of the mice were calculated. These trajectories were grouped into 20 clusters. Three clusters are displayed as examples, showing tissue-specific miRNA signatures. Cluster 2 is composed of 50.1% miRNAs originating from skin, cluster 9 of 61.2% miRNAs from brain and cluster 13 of 70.6% miRNAs from BAT. **f**, Cluster 20 of the whole organism miRNA trajectory clustering—the cluster contains two global aging miRNA, miR-29a-3p from ten different tissues and miR-29c-3p from eight different tissues.

For the intersection of miRNAs expressed in all tissues, we observed more markers being correlated positively than negatively with age (Fig. 2a and Supplementary Table 3). In contrast, large sets of miRNAs were correlated with age in a specific tissue. For example, six miRNAs were negatively correlated exclusively in limb muscle and 37 were positively correlated only in BAT. One of these miRNAs, miR-107, regulates insulin sensitivity and is postulated as a target for the treatment of type 2 diabetes and obesity^17^. Its increase in aging could be connected to the fact that age is a risk factor for diabetes^1^.

Certain miRNAs were linearly correlated with age in more than one tissue (Fig. 2b). MiR-29a-3p was positively correlated in eight tissues, and miR-300-3p, miR-487b-3p and miR-541-5p were negatively correlated in five tissues each. Based on these observations, we separated the miRNAs into the following three classes: nonaging-related, local aging and global aging miRNAs. Local aging miRNAs were defined as correlated with age in at least one tissue exceeding the interval of −0.5 to 0.5. We accordingly defined miRNAs correlated with age in more than five different tissues as globally aging. Following these definitions, we identified the three mentioned negatively correlated miRNAs together with five positively correlated miRNAs (miR-29a-3p, miR-29c-3p, miR-155-5p, miR-184-3p and miR-1895) as globally aging.

**Extended Data Fig. 4:**
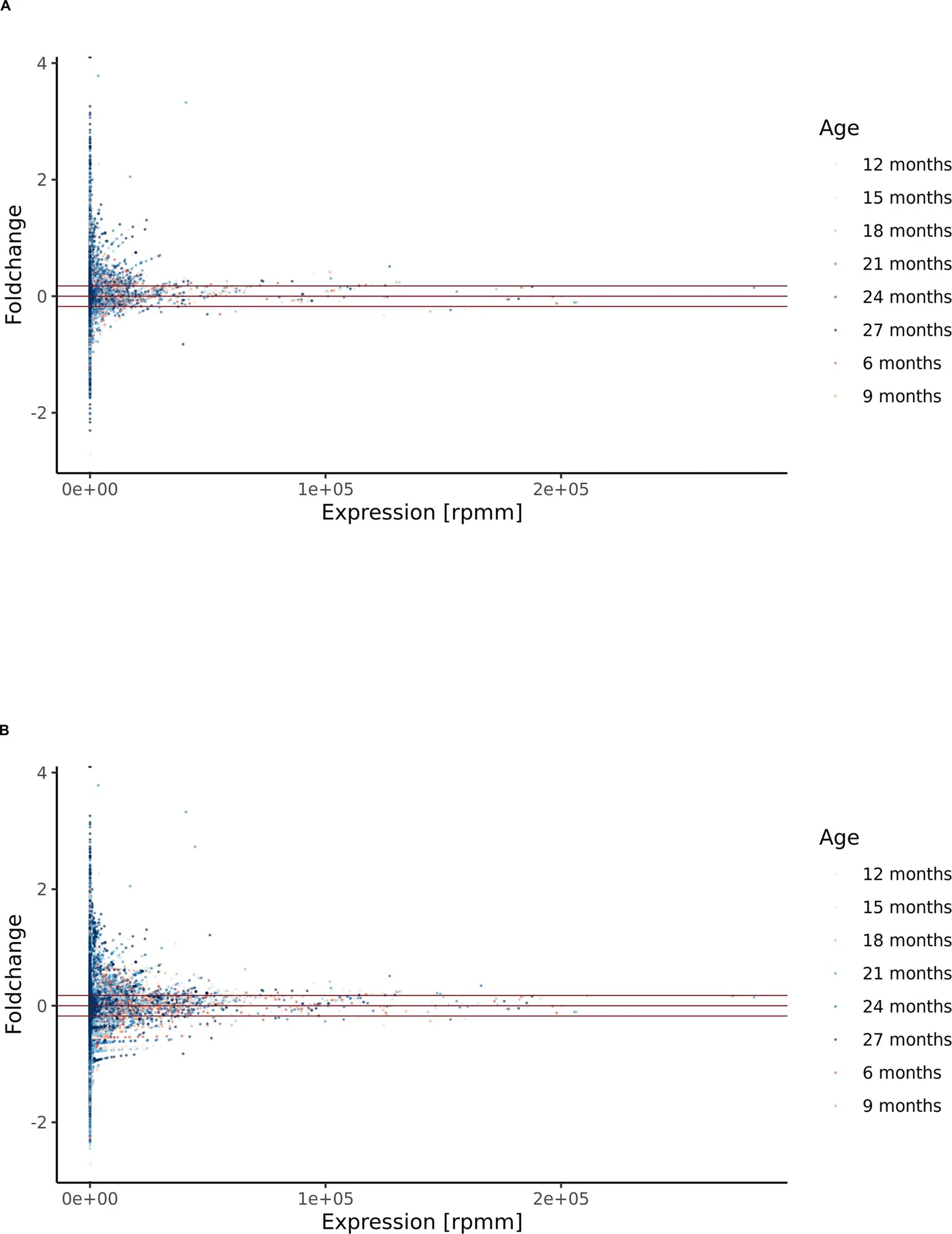
Mean expression vs. log2 FC in all tissues for all ncRNAs. Log2 foldchanges (each timepoint vs 3 months) compared to mean expression values in reads per million mapped of all non-coding RNA (A) and only miRNA (B), red lines as reference for 2/3 and 3/2, colored by timepoint (as indicated in Fig. 1a).

**Extended Data Fig. 5:**
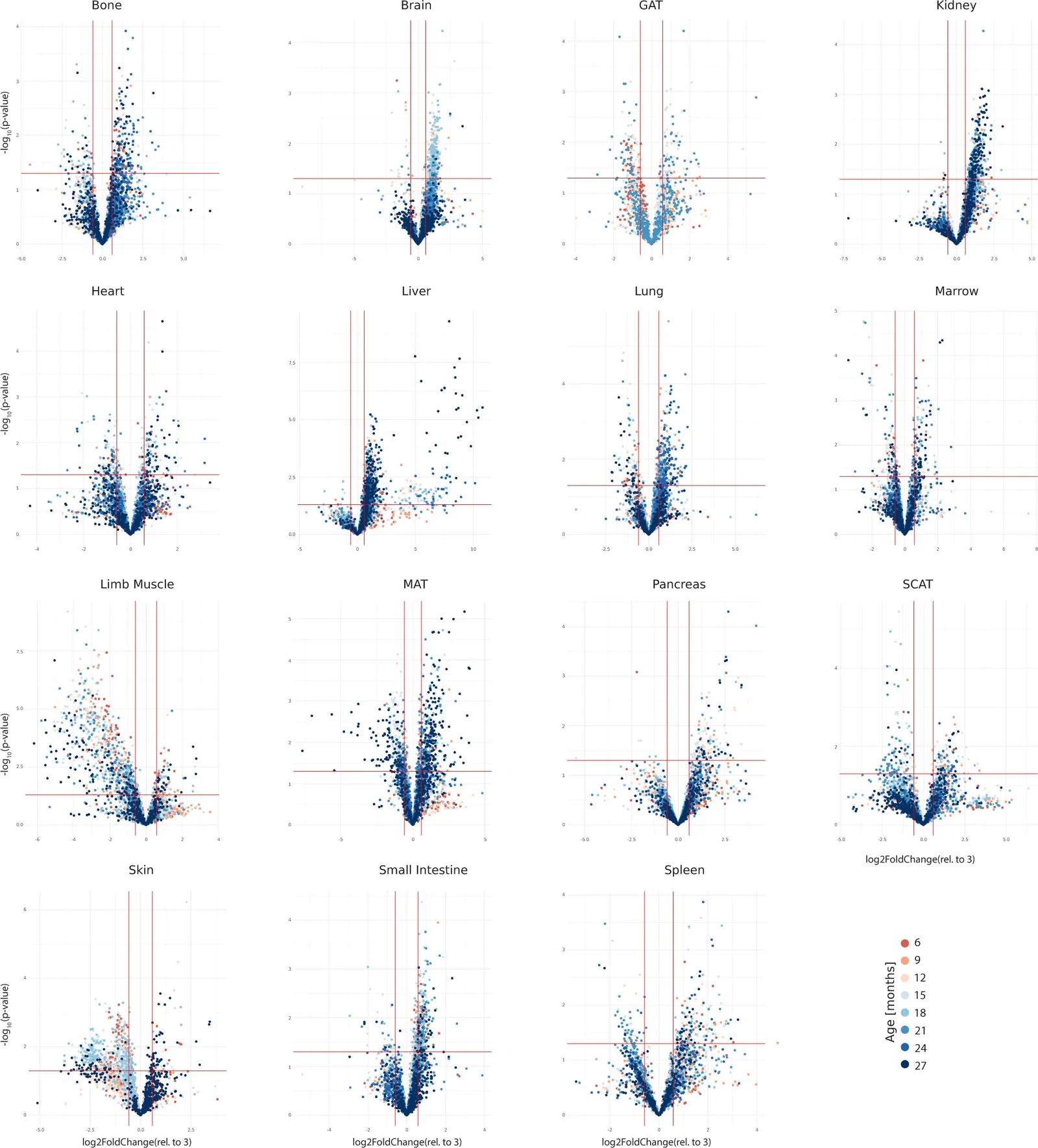
Volcano plots for comparison 3 m to all later timepoints per organ. Volcano plots for each tissue, colored by timepoints (color code as indicated in Fig. 1a). Log2(FC) are plotted versus -log10(p-values) calculated in a two-sided t-test.

We then examined whether nonlinear age-related expression changes occur as well. Using the 3m timepoint as a baseline, we calculated foldchanges (FC) for all later timepoints and respective *P* values. Based on this analysis, we determined the number of deregulated miRNAs (Fig. 2c). Most were deregulated in BAT, driven by the large fraction of positively correlated local aging miRNAs. Investigating the brain, we found a peak at the ages 12 and 18 months with a count of 412 and 427 deregulated miRNAs, respectively. Most of all substantially deregulated miRNAs (77.6%) in brain showed the strongest effect at 12 and 18 months (Fig. 2d, Extended Data Fig. 4a,b and Supplementary Table 4). The higher count of deregulated miRNA at certain timepoints matched our expectation, as we hypothesized that miRNAs were responsible for the regulation of the previously reported transcriptome changes^18^. We further confirmed that those effects were not driven by lowly expressed features—we projected the mean expression against the FC for all tissues and all ncRNAs per timepoint (Extended Data Fig. 5). In line with our assumption, substantial FCs could be observed across all expression scales.

**Extended Data Fig. 6:**
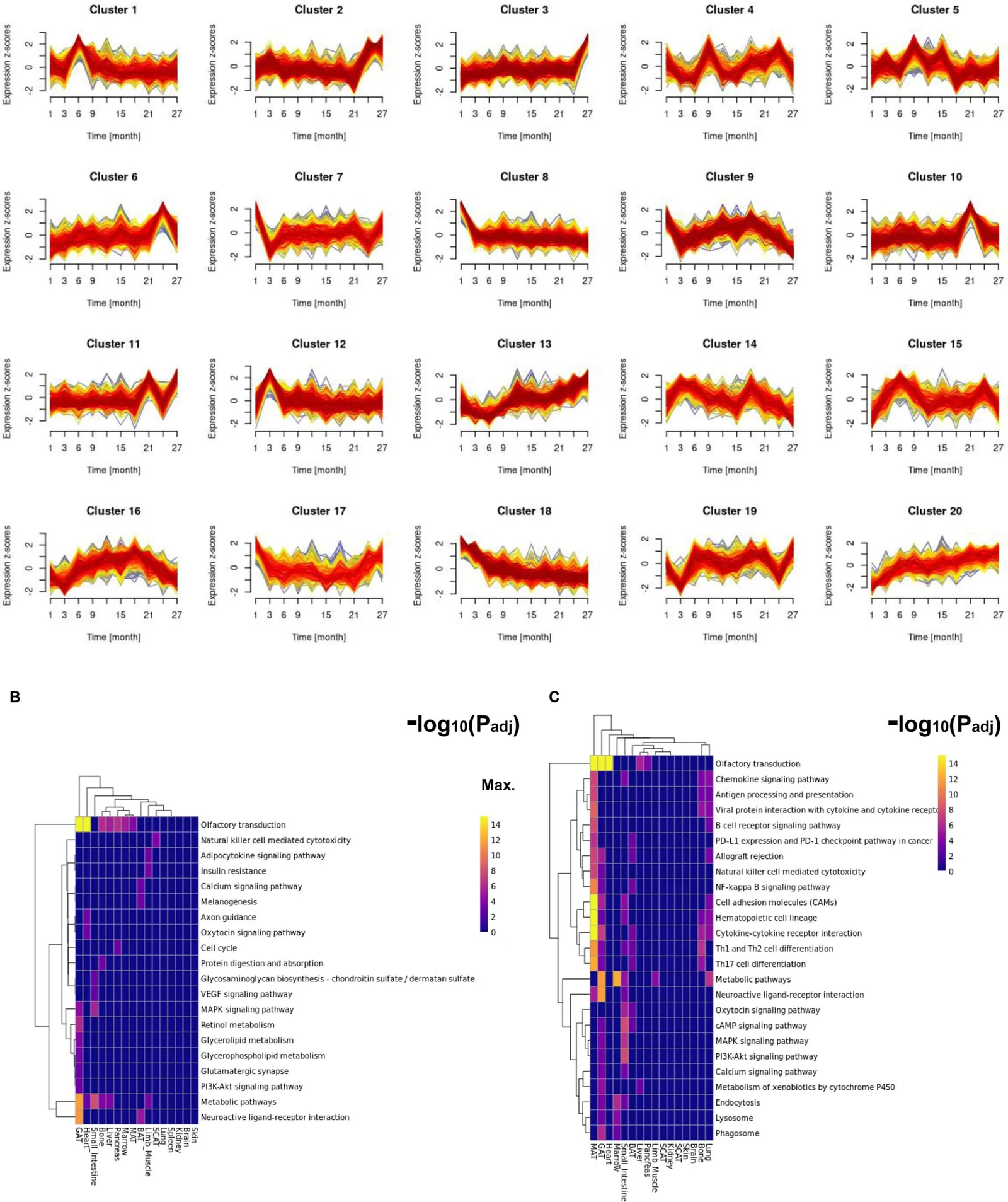
Whole organism miRNA aging trajectory clustering and enriched pathways of mRNAs targeted by local aging miRNAs. (A) Fuzzy c-means organism-wide z-scored trajectory clustering for all miRNAs in all tissues into 20 clusters. (B) Top 20 non-disease-related locally enriched significant pathways overlapping between tissues and mRNA targets identified via negative correlation with the aging miRNA set (r > 0.5, with age) in each individual tissue. (C) Top 25 non-disease-related locally enriched significant pathways overlapping between tissues. mRNA targets identified via negative correlation with miRNA correlated negatively with aging (r < -0.5) in each individual tissue (identified via overrepresentation analysis, hypergeometric test, two-sided, adjustment FDR, t-test < 0.001).

To identify common patterns within the nonlinear changes over time, we calculated *z* scores for all miRNAs being expressed in every tissue. Each miRNA in every single tissue was displayed as an aging trajectory and clustered across all organs. Ten of the 20 clusters obtained were composed mainly of one tissue; thus, we propose the existence of organ-specific miRNA time course signatures (Fig. 2e). Half of the miRNAs in cluster 2, with a peak at 3 months and a late increase again at 24 months, originated from the skin. Cluster 9, which showed a peak at 12 and 18 months, was composed of 61.2% brain miRNAs. The expression of miRNAs in cluster 13 increased continuously from the age of 6 months on, and this trajectory was specific for BAT (70.6%). In summary, we determined 10 of the 20 clusters to be tissue-specific, with at least 30% of miRNA originating from a single tissue (Extended Data Fig. 6).

The global aging miRNAs marked an exception to this tissue-specific clustering. Trajectories from more than five different organs for seven global aging miRNAs clustered together. For instance, we found the trajectories of miR-29a-3p and miR-29c-3p from ten and eight different tissues in cluster 20, respectively (Fig. 2f). The expression of miRNAs within this cluster increased continuously with age. This consistent signature could be indicative of the regulation of key pathways across all organs upon aging. Thus, we investigated the relationship between miRNA and mRNA expression closer.

### Transcriptome changes mirrored by global aging miRNAs

The previous analyses suggested five miRNAs as cross-organ aging markers increasing with age (Fig. 2b). Following the biological mechanism, we expected repression of target genes with aging. We chose to identify potential new targets in an unbiased manner by correlating miRNA with mRNA expression levels from the TMS dataset^5^. In the first step, we defined targets by exhibiting a significant inverse correlation (*r* < −0.4, *P* < 0.05). To support the validity of our approach, we checked the share of predicted miRNA–mRNA interactions with conserved binding sites for the miRNAs. As a control, we compared this number against the share of conserved binding sites in the miRNA–mRNA interactions predicted via positive correlation. For 7.3% (9 of 122) of the miRNA–mRNA interactions identified via inverse correlation, we found at least one conserved miRNA binding site, as compared to the 2.1% (120 of 54,992) miRNA–mRNA interactions in the control set (Fisher’s exact test, *P* = 0.0018). Because a gene can contain multiple binding sites across multiple 3′ UTRs and different site types exhibit different strengths, we repeated the analysis for each type of binding site. The amount of conserved 8mer binding sites is 6.3 times higher as compared to the control (4.91% inv. correlation, 0.78% control; *P* = 0.0006), for conserved 7mer-8m binding sites 3.8 times higher (4.92% inv. correlation, 1.30% control; *P* = 0.0062) and for conserved 7mer-1a binding sites 9.0 times higher (2.45% inv. correlation, 0.27% control; *P* = 0.0064).

**Fig. 3:**
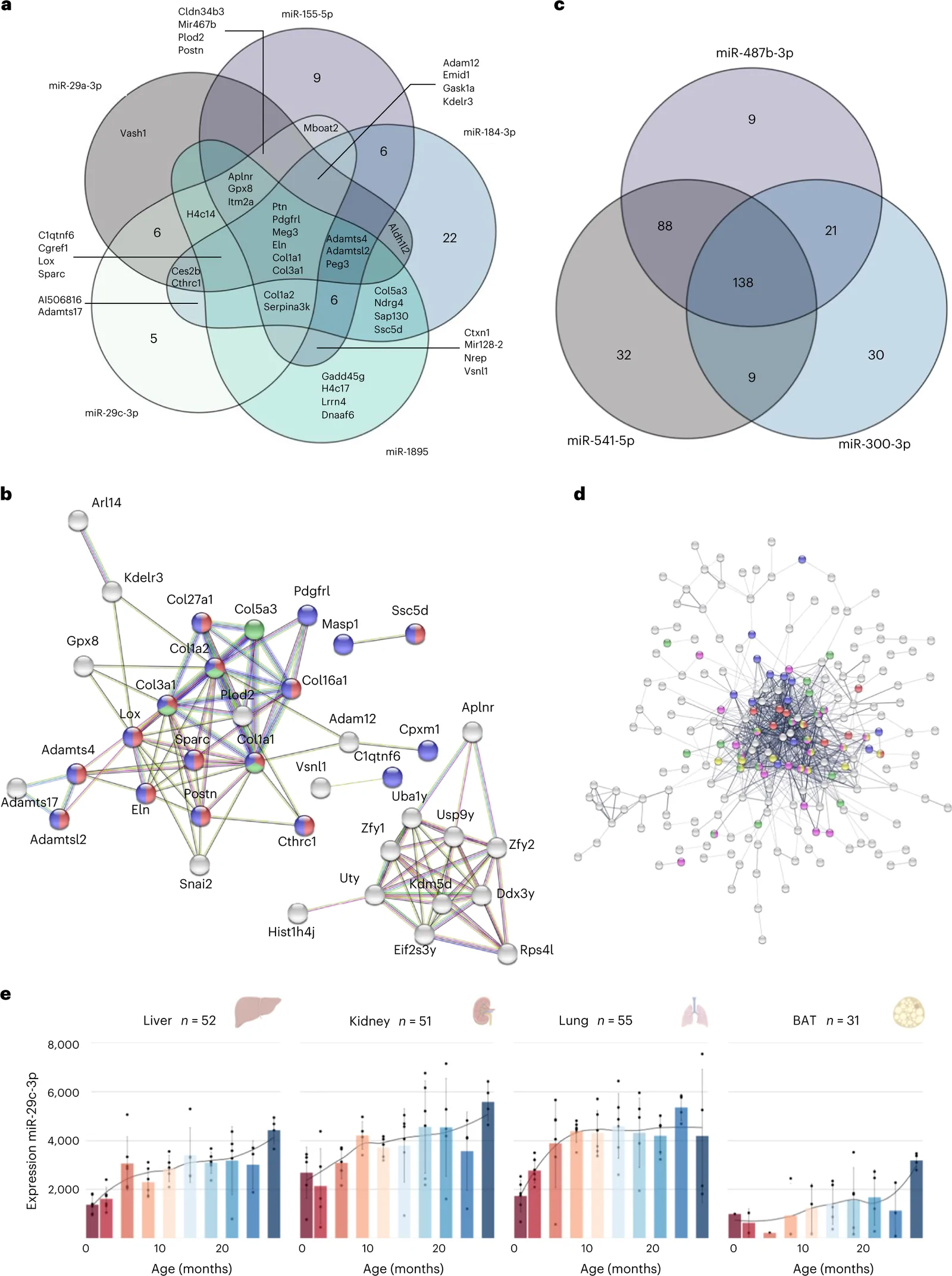
mRNA target correlation analysis for global aging miRNAs. **a**, Venn diagram of predicted target transcripts of the five global aging miRNAs positively correlated with age in most tissues. Targets are identified via inverse correlation of expression values (Spearman’s rho < −0.4, Spearman’s statistics *P* < 0.05, two-sided); only miRNA–mRNA target predictions were selected that are correlated in at least two tissues for one of the five miRNAs. **b**, STRING network for all connected proteins encoded by target transcripts of the global aging miRNAs positively correlated with age (**a**); nodes are color coded for pathways in red for ‘ECM’, purple for ‘secreted’ and green for ‘dysregulated miRNA targeting in insulin PI3K-AKT signaling’. **c**, Venn diagram of predicted target transcripts of the three global aging miRNAs negatively correlated with age in most tissues. **d**, STRING network for all connected proteins encoded by target transcripts of the negatively with age correlated global aging miRNA negatively correlated with age (**c**); nodes are color coded for pathways in red for ‘immune receptor activity’, in purple for ‘cytokine activity’, in yellow for ‘hematopoietic cell lineage’, in pink for ‘adaptive immunity’ and in green for ‘immunoglobulin’. **e**, Expression of miR-29c-3p in reads per mapped million in the liver (*r* = 0.69), kidney (*r* = 0.56), lung (*r* = 0.51) and BAT (*r* = 0.48) over the mouse lifespan (mean per timepoint ± s.d.), created with BioRender.

The filtered target gene sets showed distinct overlaps (cf. Methods; Fig. 3a and Supplementary Table 5). Three of the six targets are shared among all miRNAs, *Eln, Col1a1* and *Col3a1*, which have a role in protein digestion and absorption and encode extracellular matrix (ECM) proteins. These are already validated targets for miR-29b-1/miR-29a (ref. ^19^). Overall, enriched processes for all targets were dominated by ECM-associated processes, such as ECM organization, collagen fibril organization and ECM-receptor interaction (Fig. 3b)^20^. Senescent cells are known to exhibit altered expression and organization of ECM and the ‘senescence-associated secretory phenotype’^1,21^. Our data suggest that these effects could be regulated by global aging miRNAs. Another part of the network composed of mainly Y-chromosome-coded proteins contained proteins related to ‘ubiquitin-proteasome dependent proteolysis’ (*Usp9y*), histone modification introducing proteins (*Kdm5d*) and probable transcriptional activators (*Zfy1, Zfy2*). Hence, other layers of regulation mechanisms are targeted. The ‘AGE-RAGE signaling pathway in diabetic complications’ and ‘dysregulated miRNA targeting in insulin/PI3K-AKT signaling’ were enriched, supporting our suggestion of the importance of miRNA regulation in nutrient sensing.

Consistent with the enriched pathways for the targets of cross-organ aging miRNAs were the enriched pathways for the targets of the local aging miRNAs. The ‘PI3K-AKT signaling pathway’, ‘protein digestion and adsorption’, ‘metabolic pathways’, ‘adipocytokine signaling pathway’ and ‘insulin resistance’ were found among the top 20 locally enriched pathways in targeted mRNAs (Extended Data Fig. 6b).

Through a reduction of miRNA expression during aging, the repression of gene expression is potentially reduced or even lost. Gene targets for global aging miRNAs reducing repression with age were identified via correlation (Fig. 2b) (*r* < −0.4, *P* < 0.05). The expression of three miRNAs, miR-300-3p, miR-487b-3p and miR-541-3p, decreased during the lifespan in five tissues. The overlap of their potential targets was high, with 138 of 327 predicted interactions (Fig. 3c and Supplementary Table 6). The identified targets exhibited a functional enrichment for pathways related to immune system processes, such as ‘cytokine–cytokine receptor interaction’, ‘Th1 and Th2 cell differentiation’, ‘Th17 cell differentiation’, ‘chemokine signaling pathway’ and ‘NF-kappa B signaling pathway’. The network was particularly dense in its center, with targets related to ‘adaptive immunity’, ‘immunoglobulin’, ‘hematopoietic lineage’, ‘immune receptor activity’ and ‘cytokine activity’ (Fig. 3d). We also determined the locally enriched pathways for all miRNAs in every individual tissue whose expression decreased upon aging via inverse correlation with mRNA targets. These were similarly dominated by immune-related processes (Extended Data Fig. 6c). As immune senescence and inflammation are hallmarks of aging^1^, it is crucial to further investigate these potentially age-sensitive regulation mechanisms.

**Extended Data Fig. 7:**
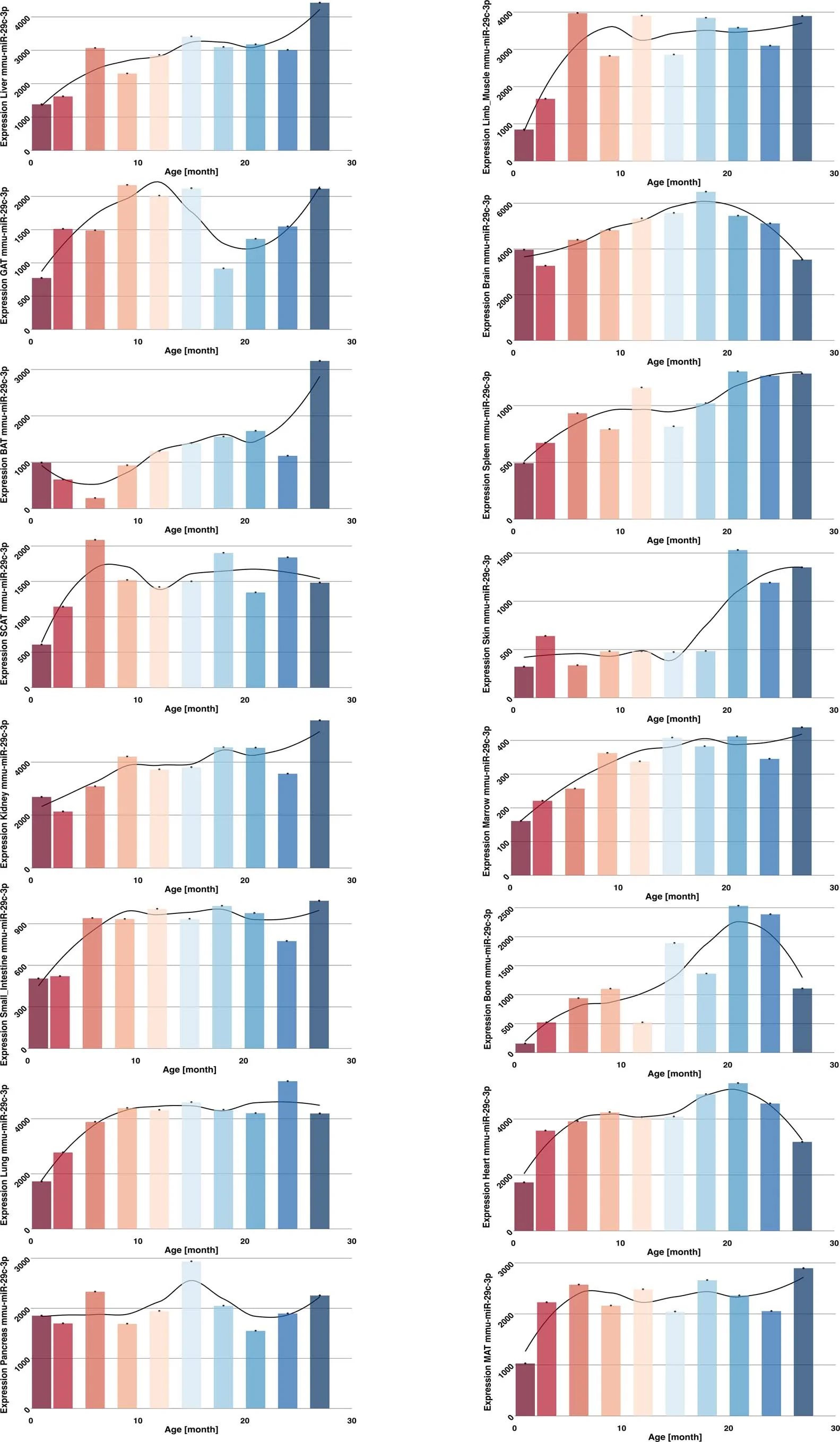
miR-29c-3p expression per tissue. Expression of global aging miRNA miR-29c-3p as reads per mapped million for all tissues at all measured timepoints.

We chose the global aging miR-29c-3p as an example for further investigation. In liver and kidney, expression increased monotonically over the lifespan as well as in BAT but at a lower baseline expression (Fig. 3e). In the lung, the steep increase during early adulthood ends at approximately 12 months of age. A general trend of miR-29c-3p expression increase was present in all tissues, but expression levels and the course of increase showed tissue-specific patterns (Extended Data Fig. 7).

### miR-29c-3p exhibits an organ-specific rejuvenation response

**Extended Data Fig. 8:**
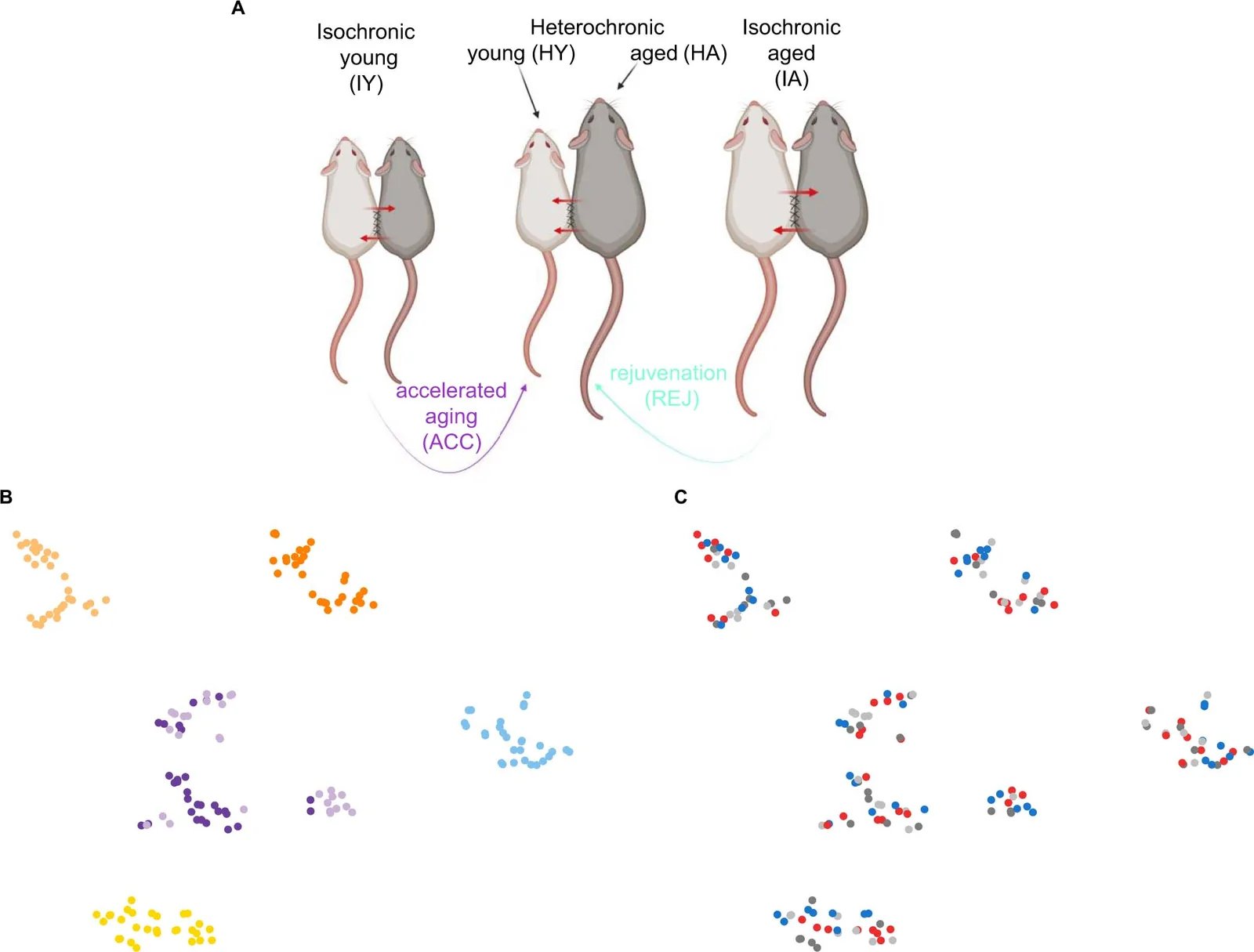
Experimental design for the parabiosis study and variance analysis of samples. (A) Schematic plot of the experimental aging intervention and heterochronic parabiosis. Visualization of all samples of the parabiosis cohort over all detected non-coding features in a t-SNE, colored by tissue (B, color code as indicated in Fig. 1a) and treatment group (C, color code as indicated in Fig. 4b).

Expansive beneficial effects on cognition, muscle strength and bone repair have been observed for heterochronic parabiosis via a shared common circulation, or systemic infusions of young blood^22^. We sequenced tissue samples from a parabiosis intervention cohort to determine whether the young blood in aged individuals influences small ncRNA expression. The cohort was composed of 176 samples from six different organs of isochronic young (IY) and aged (IA), and heterochronic young (HY) and aged mice (HA) (Supplementary Table 1). Rejuvenation, the reversion of aging aspects, is the desired outcome of the intervention. However, it is accompanied by accelerated aging, the negative effect of the young sharing their blood with the old. In our study, the rejuvenation effect was measured by comparing the expression levels in IA mice with those detected in HA mice. In turn, the accelerated aging effect was defined by the difference between IY and HY mice (Extended Data Fig. 8a). Healthy aging was defined as the comparison of mice from the TMS cohort aged 3 and 21 months (AGE), closely matching the age distribution of the parabiosis cohort at takedown. Clustering the samples using t-SNE revealed tissue identity as major driver of variance across the experimental groups (Extended Data Fig. 8b,c).

**Fig. 4:**
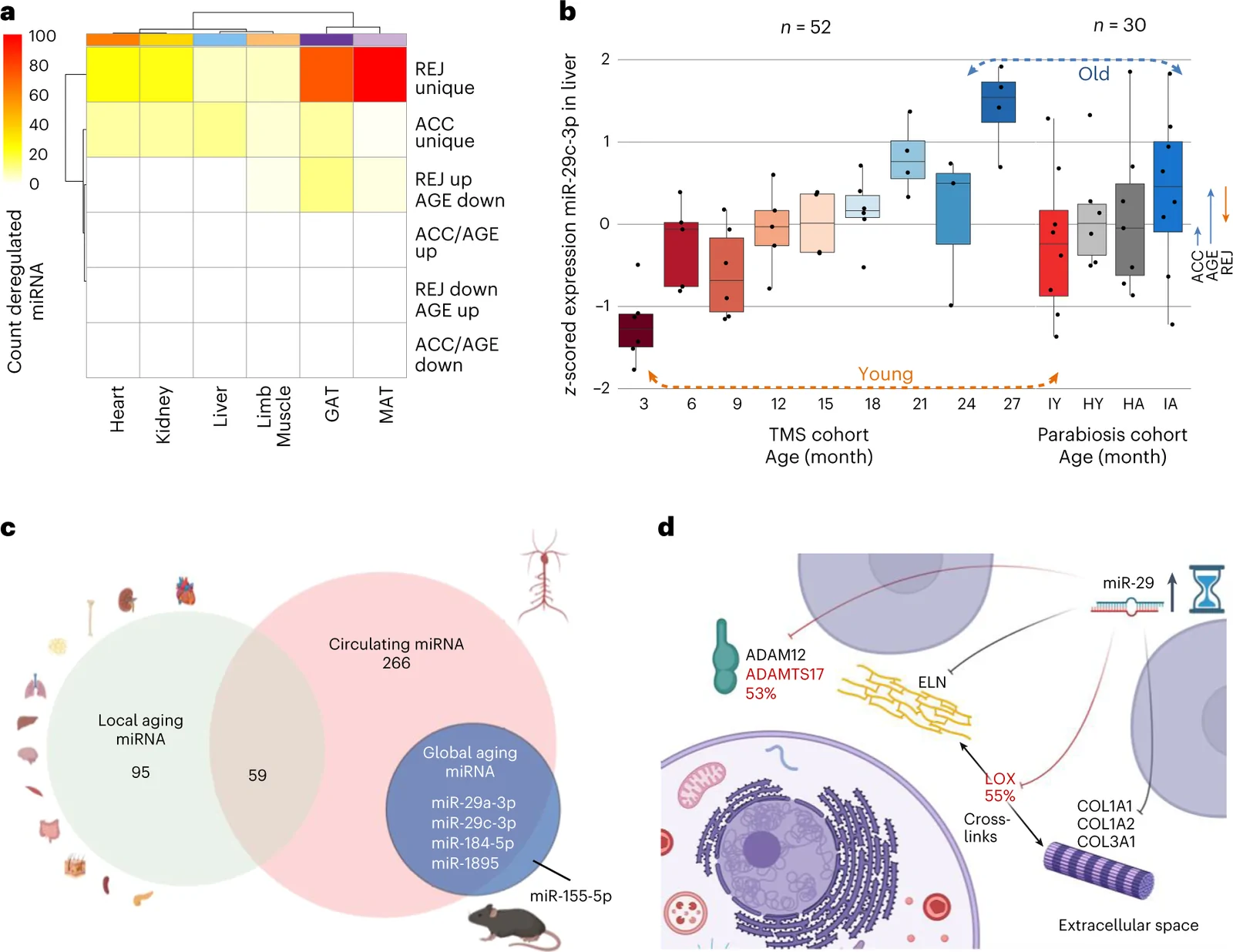
Compiled systemic mechanisms of global aging miRNAs. **a**, Heatmap of the absolute numbers of deregulated miRNAs in parabiosis for the six comparisons in all analyzed tissues—uniquely deregulated in REJ, uniquely deregulated in ACC, in REJ up-regulated and healthy aging (AGE) downregulated, and vice versa, in AGE and ACC up-regulated or downregulated, all values >100 set to 100. **b**, Boxplot of expression *z*-scores for miR-29c-3p per timepoint in the liver for healthy aging from the TMS cohort and *z*-scores of miR-29c-3p in the liver from the parabiosis cohort for the following four different groups: IY, HY, HA and IA; arrows on the right indicate the effect size of aging in the parabiosis cohort, rejuvenation and accelerated aging. Boxes span the first to the third quartile with the median value represented as line inside the box. The whiskers show the maximum and minimum values or values up to 1.5 times the interquartile range above and below the first or third quartile if outliers are present. **c**, Venn diagram of local aging miRNAs (exclusively positively correlated with age in one tissue; green), circulating miRNAs (all miRNAs expressed in EVs and serum, red) and global aging miRNAs (positively correlated with age in more than five tissues, blue). **d**, Summary of validated targets of miR-29 family members, shown in their cellular location. These targets are key proteins in processes related to the ECM; newly validated targets are shown in red. Created with BioRender.

We assigned deregulated miRNAs to the following groups: either (1) uniquely deregulated in rejuvenation (REJ unique) or in accelerated aging (ACC unique), or (2) deregulated in physiological aging as well as rejuvenation (REJ up and AGE down, REJ down and AGE up) or accelerated aging (AGE and ACC up/down). We found 233 uniquely deregulated miRNAs in rejuvenation and 43 in accelerated aging (Fig. 4a). Intriguingly, 17 age-related miRNAs were deregulated in the opposite direction in REJ. No miRNAs were deregulated in AGE and in the same direction in ACC, but the uniquely rejuvenated miRNAs were enriched in certain pathways in MAT (‘insulin resistance’, ‘adipocytokine pathway’, ‘type 2 diabetes mellitus’), which again have a role in nutrient sensing.

**Extended Data Fig. 9:**
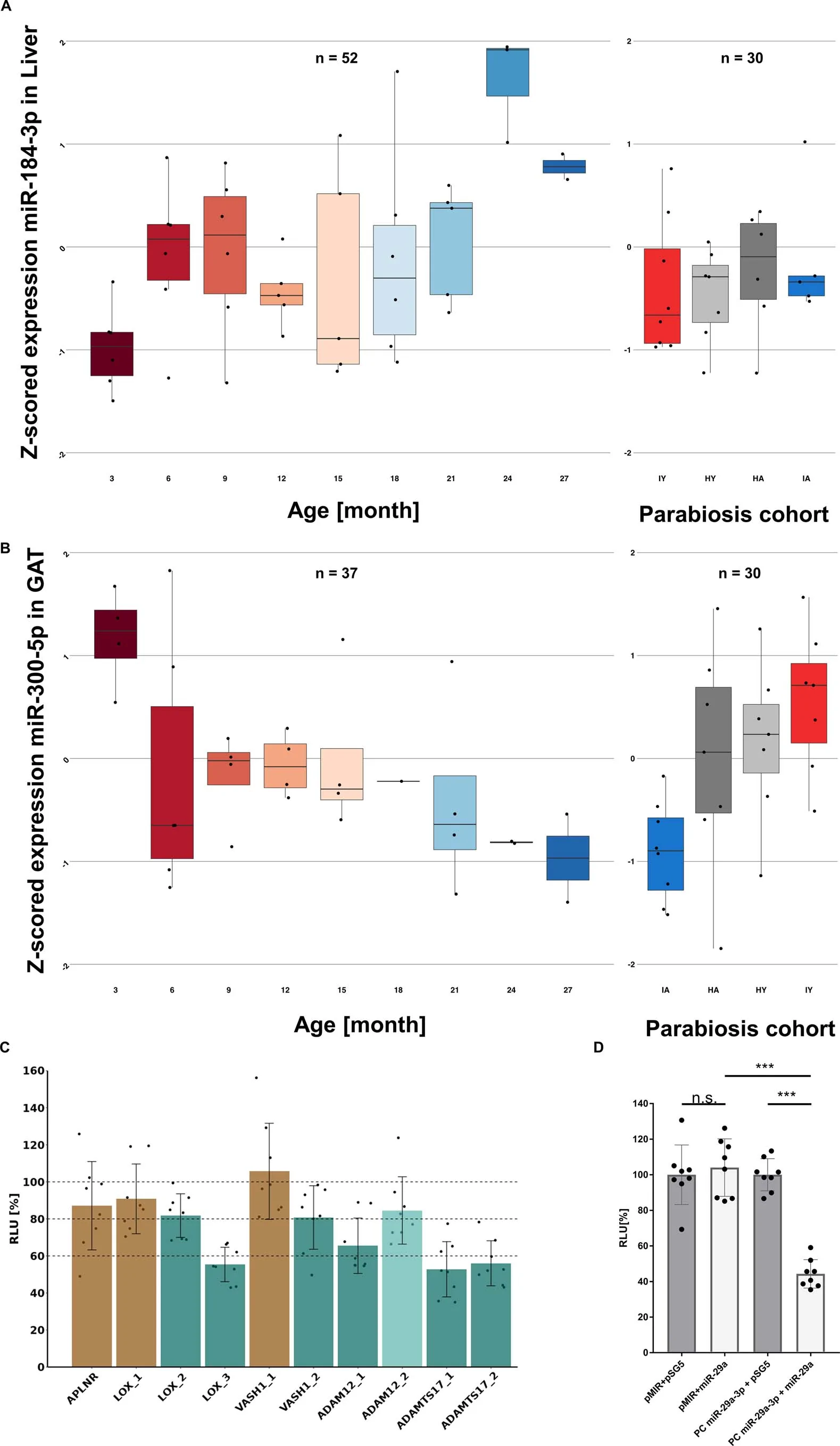
Observed rejuvenation effects of global aging miRNAs and validation experiments for predicted targets of miR-29c-3p. z-scored expression in the healthy aging cohort (TMS) for every timepoint and z-scored expression in the parabiosis cohort for the four different groups (IY, HY, HA and IA) for miR-184-3p in the liver (A) and miR-300-5p in GAT (B). The first to the third quartile are covered within the boxes with the median value shown as line inside the box. Maximum and minimum values are shown as whiskers or values up to 1.5-times the interquartile range above and below the first or third quartile if outliers are present. (C) Measured RLU (relative light unit) in percent for all target constructs for miR-29; reduction to under 80% determines a high confidence target, reduction between 80-90% low confidence target (n = 4 biologically independent experiments for each target, each as technical duplicates). Color coded in dark green for significant reduction with a p-value ≤ 0.01 (ADAMTS17_1: 0,0000004, ADAMTS17_2: 0,00000007, ADAM12_1: 0,00001, LOX_2: 0,0006, LOX_3: 0,000000001, VASH1_2: 0,0067), light green for significant reduction with a p-value ≤ 0.05 (AD-AM12_2: 0,0306) and in light brown for non-significant reduction with a p-value ≥ 0.05 (n.s; APLNR: 0,1485, LOX_1: 0,1892, VASH1_1: 0,5457). P-values were calculated by an unpaired t-test. Data are shown as mean ± SD. (D) Control experiment results luciferase assay, negative control: vector alone and vector with miRNA and positive control with vector (pMIR+ pSG5; pMIR+miR-29a, PC miR-29a-3p + pSG5) and positive control (PC miR-29a-3p + miR-29a) (n = 4 biologically independent experiments, each as technical duplicates; *** = p-value ≤ 0.001, n.s. = p-value ≥ 0.05). P-values were calculated by a Welsh’s t-test (pMIR+pSG5 vs. pMIR+miR-29a = 0,6314; PC miR-29a-3p+miR-29a vs. PC miR-29a-3p + pSG5 p-value < 0,0001; PC miR-29a-3p+miR-29a vs. pMIR+miR-29a p-value < 0,0001.) Data are shown as mean ± SEM.

For three global aging miRNAs, we discovered that changes in expression observed during healthy aging can be partially reversed in response to parabiosis. For miR-29c-3p, we measured a strong rejuvenation effect in the liver, four times higher than the effect of accelerated aging (Fig. 4b). The other two global aging miRNAs miR-184-3p in the liver and miR-300-5p in GAT showed similar trends of reversed expression but with a lower magnitude (Extended Data Fig. 9a,b). Considering the pronounced globally aging versus local rejuvenation profile of miR-29c-3p, we chose to explore systemic effectors and mediators of these signals.

### Expression of circulating mir-29 family increases with aging

MiRNAs can circulate in the plasma and EVs between organs. We thus assessed the abundance of miR-29c-3p in both plasma and the vesicle-bound fraction using an independent cohort^23^. Analyzing the expression at five timepoints across the lifespan from 2 to 18 months allowed us to correlate and compare the abundance of the miRNA in plasma and EVs. We observed an increase of miR-29c-3p expression correlated with age for both fractions (*r* = 0.56 (plasma) and 0.65 (EVs)). The share of positive global aging miRNAs detected as circulating was higher than the share of local aging miRNAs (38.3%) (Fig. 4c). This supports our hypothesis that miRNAs traveling via the shared circulatory system could have a role in the positive effect of parabiosis.

MiR-29c-3p could regulate gene expression in pathways resulting in health improvements by entering the tissue via the blood in vesicles. Recently, miRNAs with certain sequences were shown to be more likely secreted in small EVs, and their capability to inhibit target genes in recipient cells is enhanced^8^. One so-called EXOmotif is CNGGNC, which is very similar to a sequence found in the mature mmu-miR-29c-3p CUGGUG. We performed luciferase assay experiments to validate our predictions for mir-29-family members on target genes related to aging. *Lox* and *Adamts17* were validated as high confidence targets, and *Vash1* was validated as a low confidence target (Extended Data Fig. 9c,d). Previously known targets from the literature (*Eln, Col1a1, Col1a2, Col3a1 and Adam12*), as well as *Lox* and *Adamts17*, are components in ECM processes (Fig. 4d), supporting our hypothesis that mir-29-family members have a crucial role in organismal aging due to their repressive regulatory function on these targets.

## Discussion

We extended the TMS and parabiosis transcriptome datasets by bulk ncRNA sequencing and combined the data to highlight interactions of biomolecules and their functions to reveal potential regulatory mechanisms of aging. We report organ-specific trajectories during aging for miRNAs using organism-wide clustering. We thereby observed enrichment of miRNAs in pathways related to insulin resistance, especially for adipose tissue organ-specific miRNA trajectories. These results relate the miRNA expression changes to deregulated nutrient sensing.

Moreover, we identified global aging miRNAs negatively and positively correlated with age. The increased expression levels of miR-29c-3p in age are partially reversible through heterochronic parabiosis. miR-29c is known as a negative regulator of RAG1 in B cells in mice and humans. Overexpression of miR-29c thereby reduces V(D)J recombination^24^, which is a major process shaping the immune system repertoire, to support clearance of infectious agents, infected cells and cells on the verge of malignant transformation^1^. The global increase in this miRNA in several tissues and the already-known regulation of the immune system suggest that the immune senescence aggravating the aging phenotype could be caused by this development. Another age-related pathology is the process of cellular senescence, which is regulated by the TGF-β/Smad pathway. TGF-β signaling involves miR-29-induced loss of H4K20me3 to promote senescence^25^. In a brain-specific miR-29 knockdown mouse, sex-specific effects on lifespan and reproduction were observed^26^. To prove that miR-29 has a causal role in processes responsible for cellular aging and rejuvenation, detailed knockdown or knockout experiments are needed.

**Extended Data Fig. 10:**
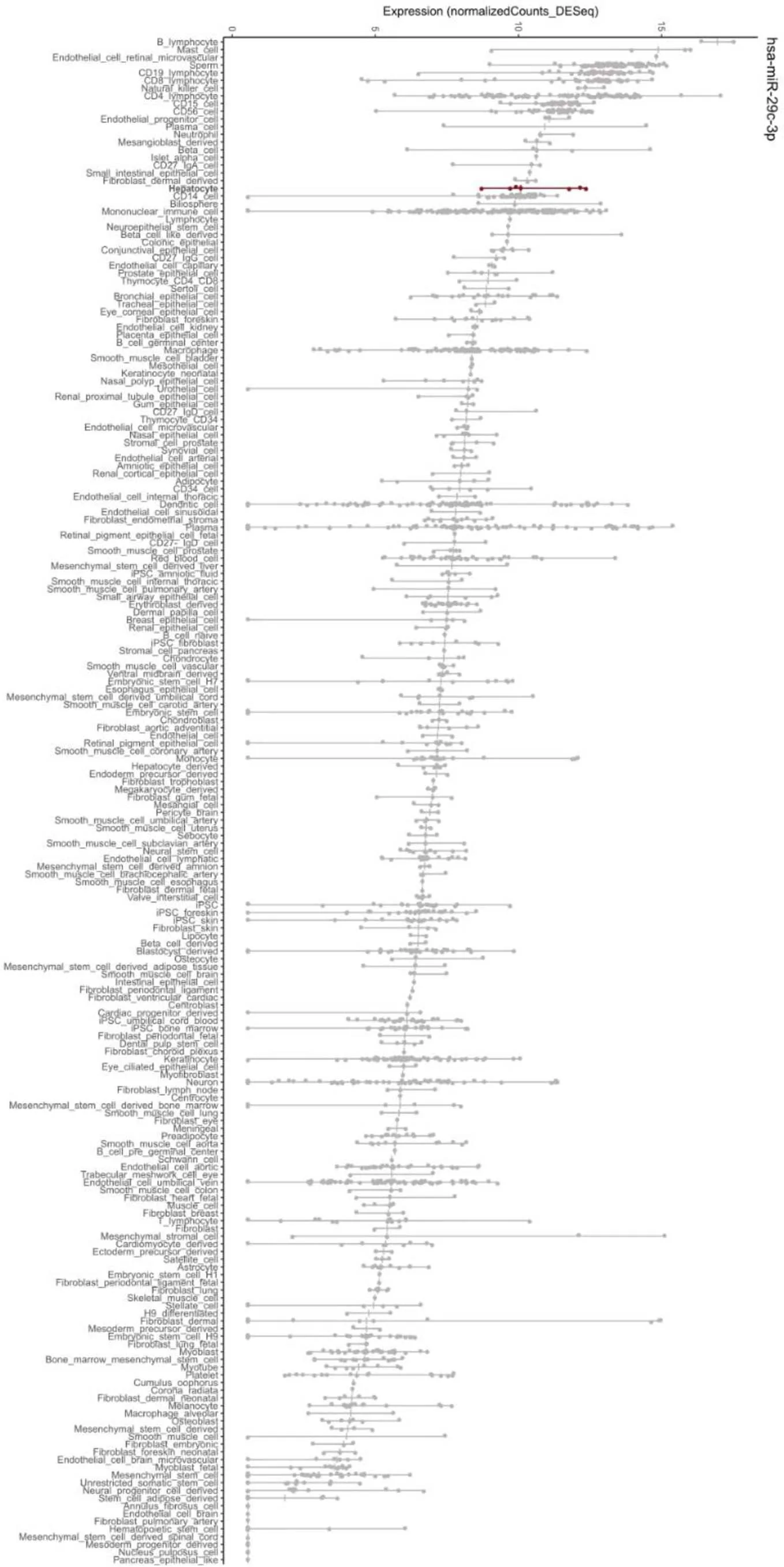
Human miR-29c-3p expression in cell lines. Expression of hsa-miR-29c-3p in human cell lines as normalized DESeq2 counts, Hepatocytes highlighted in red, data re-analysis from publication.

Future studies should also focus on gathering single-cell miRNA data to explain which cell types are responsible for the expression of miRNA aging markers. High-throughput single-cell sequencing and vesicle sequencing could help us to distinguish between cellular miRNA expression and vesicles. MiRNAs can be transported via EVs and thereby mediate the regulation of aging-related processes^27^. Also, miRNA-mediated gene silencing, which we based our targeting analysis on and used for validation, is only one mode of action of gene expression regulation. Other modes of action worth mentioning are miRNA-mediated translational activation, miRNA-mediated transcriptional and post-transcriptional gene regulation within the nucleus^28^. A more detailed view of cell-type-specific and vesicular expression might explain why we found distinct miRNA trajectories of aging in adipose tissue while the strongest rejuvenation effects for global aging miRNAs, especially miR-29c-3p, occurred in the liver. The miRNA is known the be expressed highest in T and B cells^24^, but is also expressed in liver hepatocytes (Extended Data Fig. 10) and reported as a potential tumor suppressor in human^29,30^. Hence, revealing the responsible cell type could help illuminate which mechanisms modulate miRNA expression levels. It is also necessary to discern which changes impact the transcriptome and proteome in different tissues and cellular compositions, as miRNA targetomes can differ across cell types^31^. Currently, only a few different protocols for single-cell miRNA sequencing exist and no high-throughput gold standard is available^32^.

Another limitation of the study is the known issues of small RNA library production as adapter ligation bias, adapter dimer contamination, polymerase chain reaction (PCR) amplification bias, barcode bias and the influence of RNA degradation on ncRNA profiles^33–35^. The challenge of sequencing mainly fragments for six of the eight RNA classes is related to these. Potentially, a major part of piRNA reads in the somatic tissues could have been derived from piRNA-sized fragments of other ncRNAs. These fragments are annotated in piRNA databases even though their biogenesis is perhaps independent of the PIWI pathway^13^. However, these piRNA-like small RNA are known to have important roles outside of the germline^36^. TRNA-derived small RNAs, which have a biological role by inhibiting translation or regulating gene expression, are studied likewise in aging and age-related diseases^37^. We decided not to exclude these data, so our study can be used as a reference for future studies aiming to analyze for instance tRNA-derived fragments or piRNA-like small RNAs in more detail. Of note, the fragments of longer ncRNAs are not necessarily surrogates of the full-length mature transcripts but can occur due to degradation processes. The biological function of respective mapping results remains to be explored.

In summary, our study provides a rich resource for biologists across many disciplines, as ncRNAs for all major organs across the entire lifespan of the mouse were sequenced. Reference data for healthy aging are important, because miRNAs are promising candidates for age-specific disease biomarkers^10^, and patterns of physiological aging must be defined not only in blood but also in every solid organ to promote the development of successful RNA-based therapies.

## Methods

### Samples

Mouse tissues of the aging cohort were obtained, and RNA was isolated as previously described^5^. From the National Institute on Ageing colony (Charles River) male and virgin female C57BL/6JN mice were shipped to the Veterinary Medical unit at the VA Palo Alto. The mice were housed on a 12-h light/dark cycle at 20–24 °C with food and water provided ad libitum. Humidity was monitored daily and between 23% and 55%. Mice from both cohorts were anesthetized with 2.5% vol/vol avertin, and mice were weighed and shaved. Blood was drawn via cardiac puncture before transcardial perfusion with 20 ml PBS. Dissection of organs was performed in the following order and then instantly frozen on dry ice: pancreas, spleen, brain, heart, lung, kidney, mesenteric adipose tissue, intestine (duodenum), gonadal adipose tissue, muscle (tibialis anterior), skin (dorsal), subcutaneous adipose tissue (inguinal pad), brown adipose tissue (interscapular pad), bone and bone marrow (femurs and tibiae). Bulk RNA samples of the heterochronic parabiosis cohort consisting of male C57BL/6JN, C57BL/6J and C57BL/6-Tg(UBC-GFP)30Scha/J mice were collected as previously described^22^ (Supplementary Table 1). The 3- to 4.5-month-old and 19-month-old mice were housed under the same conditions as the aging cohort mice. Suturing together the peritoneum of adjacent flanks of two mice, forming a continuous peritoneal cavity, accomplished the aging intervention parabiosis via the peritoneal method. To enable coordinated movement after surgery, adjacent knee joints and elbow joints were joined with nylon monofilament sutures, as well as skin with surgical autoclips. These procedures were conducted with aseptic conditions on heating pads, with mice under continuous isoflurane anesthesia. Mice were injected with Baytril (5 µg g^−^^1^), buprenorphine and 0.9% (wt/vol) sodium chloride to avoid infection, limit pain and promote hydration, as previously described in ref. ^22^. For 5 weeks, the pairs remained together, and organs were collected. First, heart, liver, kidney, then MAT and GAT, and finally limb muscle were collected in this order, all within 30–40 min. All animal care and procedures were carried out in accordance with institutional guidelines approved by the VA Palo Alto Committee on Animal Research (Protocol, LUO1736). RNA was isolated according to the manufacturer’s protocol with the miRNeasy Kit (Qiagen, 217084). All RNA samples were shipped to the Institute of Human Genetics. Samples of the TMS cohort were additionally precipitated due to salt contamination. In brief, 150 ng of RNA was mixed with 3 M NAAC (pH 7.0) and 100% EtOH and incubated overnight at -20 °C. This was centrifuged at 20,817*g* at 4 °C for 60 min. Supernatant was discarded and the pellet was washed with 80% EtOH, followed by another centrifugation for 30 min (20,817*g*, 4 °C). Supernatant was again discarded, the pellet was dried on ice and resuspended in 50 µl 1x TE buffer. Quality control of concentration was performed with the NanoDrop 2000 spectrophotometer (Thermo Fisher Scientific), and the RNA integrity was determined using the Agilent RNA 6000 Nano Kit (Agilent Technologies, 5067-1512) for randomized samples of the cohorts.

### Sample size, randomization and blinding

No sample size choice was performed before the study. During mouse dissection, order and preparation of 96-well plates for cDNA creation randomization was performed. No blinding was performed; the authors were aware of all data and metadata-related variables during the entire course of the study.

### Library preparation

Small RNA library preparation was performed using the MGIEasy Small RNA Library Prep Kit (Item 940-000196-00) and the high-throughput MGI SP-960 sample prep system according to the manufacturer’s protocols. In brief, 3′- and 5′-adapters were ligated to the RNA, and reverse transcription (RT) was performed using an RT primer, in which sample-specific barcodes were incorporated. The resulting cDNA was amplified in a PCR with 21 cycles. The amplification product was size selected and purified using AMPure Beads XP (Beckman Coulter). The size of the purified PCR products was checked using an Agilent DNA 1000 Kit (Agilent Technologies), and the concentration was determined using Qubit 1X dsDNA High Sensitivity (Thermo Fisher Scientific). For each library, 16 samples, barcoded with barcodes 1–4, 13–16 and 25–32, were pooled in an equimolar fashion at a concentration of 4.56 ng µl^−1^. Pooled libraries were circularized and sent for sequencing. A total of 65 libraries consisting of 947 samples were analyzed in the project.

### Sequencing and data analysis

Samples were sequenced single-ended on the BGISEQ500RS using the High-throughput Sequencing Set (SE50) (Small RNA) as a service provided by BGI. The sequencing data were processed with miRMaster 2.0 using standard settings^38^ and mapped read percentages were generated. Data analysis was performed using RStudio Software v4.0.3 with the following packages: viper v1.26.0, data.table v1.14.2, ggrepel v0.9.1, ggvenn v0.1.9, M3C v1.14.0, ggridges v0.5.3, forcats v0.5.1, purrr v0.3.4, tidyr v1.2.0, tibble v3.1.6, ggplot2 v3.3.5, tidyverse v1.3.1, viridisLite v0.4.0, ColorBrewer v1.1-2, reshape2 v1.4.4, pheatmap v1.0.12, Mfuzz v2.52.0, DynDoc v1.70.0, widgetTools v1.70.0, e1071 v1.7-9, stringr v1.4.0, dplyr v1.0.8, readr v2.1.2 and Biobase v2.52.0.

Samples were excluded if fewer than 2 million aligned reads were detected while allowing one mismatch per read. Using Bowtie (v1.2.3.), reads were mapped against the RNA sequence derived from the respective databases (miRNAs: miRBase 22, tRNAs: GtRNAdb 18.1, piRNA: RNACentral 15, all other ncRNAs: Ensembl 100). Only the first paralog was retained for analysis, additional paralogs are listed in Supplementary Table 7. As the lengths of the mature ncRNAs matched with our sequencing read length exclusively for miRNAs and piRNAs^39^, we calculated detailed covered sequence length statistics. These analyses verify that not only random fragments were sequenced for the other ncRNA classes. Such fragments can occur as a result of a physiological process like tRNA-derived fragments and have a regulatory role in aging^37^ or can be products of postmortem RNA degradation. The amount and distribution of degradation fragments are highly influenced by the RNA quality^35^. Covered read length, reference read length, longest covered region, covered percentage reference length, longest mapping read, total reads mapping and average covered read length are listed in Supplementary Table 2 for all detected ncRNA. All mature ncRNAs are represented by their highest counting precursor.

Percentages of aligned reads per sample derived from the miRMaster analysis were used to calculate mean percentages within each tissue and each timepoint. As a first filtering step, piRNAs were filtered for those encoded in prepachytene genomic piRNA clusters as an established method to identify true somatic piRNAs^14,15^.

For global analyses (analyses independent of the organ), samples were filtered for 1 rpmm in at least one sample in the cohort. As a global analysis, we performed a t-SNE and a principal variance component analysis (PVCA). All samples were clustered in an unweighted t-SNE with a seed set to 40 using the M3C package. A t-SNE is an optimized dimensionality reduction method used for the visualization of high-dimensional data^40^. A PVCA was used to estimate the variability of biological and technical parameters. Data dimensionality is reduced through a principal component analysis.

For local analyses, tissue-specific patterns were considered, further requiring that ncRNAs were expressed with 1 rpmm in at least 10% of the samples for each tissue. Percentages of counts per RNA class and tissue were calculated with the total number of counts within a tissue and the respective mean number of counts of the RNA classes. Percentages of counts per tissue and timepoint were calculated with the percentage of counts per sample after local filtering with the total RNA class counts. Timepoint percentages were calculated as means of the corresponding samples at each timepoint and in each tissue. Spearman rank correlations with age of each ncRNA expressed in each tissue were calculated and illustrated in a density plot grouped by RNA class. Spearman rank correlation between miRNA expression and age was categorized into positively (*r* > 0.5) and negatively (*r* < −0.5) correlated and annotated with *P* values (Supplementary Table 8). Based on this categorization, the number of tissues in which a miRNA was (anti-) correlated with age was determined. MiRNA FCs in each tissue were computed with the mean expression of each later timepoint, always comparing against 3 months. Features with mean expression of 0 for 3 months of age were excluded from this analysis. FCs lower or higher than 2/3 and 3/2, respectively, were considered deregulated. *P* values were only calculated with *t*-tests for comparisons with at least three samples per timepoint and adjusted for each tissue and timepoint separately with the Benjamini and Hochberg method. For the volcano plots, log_2_(FC) was calculated and all FC equal to 0 were discarded. Volcano plots for each tissue were generated with the −log_10_(*P* values) versus the log_2_(foldchange) and colored by timepoint. Organism-wide miRNA trajectory clustering was performed using the Mfuzz package, which clustered based on fuzzy c-means algorithms, and the number of clusters c between 2 and 20 was individually determined for each clustering using the minimum centroid distance measure. For the organism-wide clustering of the *z*-scored miRNAs over all tissues, 20 was determined as optimal. A cluster was deemed tissue-specific if at least 30% of the miRNAs in a cluster were tissue-specific.

The coding transcriptome data for the same samples were obtained from the previous study^5^. mRNA targets of miRNAs were identified via negative correlation (*P* < 0.05, *r* < −0.4). For the local miRNA–mRNA interaction analysis, miRNAs exceeding the age-correlation interval between −0.5 and 0.5 were considered. The more stringent filtering approach for aging miRNAs was chosen to discover a small set of strong candidates from the millions of possible miRNA–mRNA interactions. For the global analysis, we considered targets inversely correlated with either the positive global aging miRNAs (miR-29a-3p, miR-29c-3p, miR-155-5p, miR-184-3p and miR-1895) or the negative global aging miRNAs (miR-300-3p, miR-487b-3p and miR-541-5p) in at least two tissues, to obtain the filtered target gene set. Using STRING, the protein–protein association network database^20^, we illustrated known connections between proteins encoded by the predicted mRNA targets of the global miRNAs.

For pathway enrichment analysis, an overrepresentation analysis (ORA) was performed with the target mRNAs of global miRNAs using GeneTrail 3.2 (ref. ^41^). An ORA was performed to identify the pathways negatively and positively regulated locally in all tissues through the local aging miRNAs. The standard parameters were used, with FDR adjustment and 0.001 as significance level. Heatmaps for positive and negative regulation of miRNA on target mRNA expression were generated with the top 20 and 25 nondisease-related pathways, respectively, (lowest *P* values) regulated in most tissues.

For the parabiosis cohort, sequenced samples were analyzed with the same filtering criteria as TMS samples. We detected 50,776 ncRNAs in the raw reads of this cohort and we filtered 5,248 abundant ncRNAs for the global analysis (t-SNE). To quantify the effects of parabiosis in each tissue, FCs were calculated between IY and HY mice for the effect termed accelerated aging (ACC) and between IA and HA mice for the effect termed REJ. The effects of physiological aging (AGE) were defined as the FC between 3- and 21-month-old mice from the TMS cohort, corresponding to the ages of young and aged mice in the parabiosis experiment. As previously, FCs exceeding the interval of 2/3 and 3/2 and a significant *P* value (*P* < 0.05) were considered as deregulated.

Expression data from the EVs study were obtained as previously reported in ref. ^23^. miRNAs were considered as expressed if they were detected with at least 1 rpmm for more than 10% of the samples of one group. The intersection between miRNAs expressed in circulating plasma and EVs and either global or local aging miRNAs was determined and visualized as a Venn diagram.

### Cell lines

The HEK 293T (ACC 635) was purchased from the German collection of microorganisms and cell cultures (Deutsche Sammlung von Mikroorganismen und Zellkulturen, DSMZ). STR fingerprinting by DSMZ confirmed the authenticity of the cell line. The cells were cultivated with DMEM (Life Technologies) supplemented with Penicillin (100 U ml^−^^1^), Streptomycin (100 µg ml^−1^) and 10 % (vol/vol) FCS and passaged two times a week for not longer than 3 months.

### miRNA expression plasmid and 3’UTR reporter plasmids

The cloning of the pSG5-miR-29a expression plasmid was described previously^42^. Targets for reporter plasmids were selected based on the predicted target genes for miR-29 from Fig. 3a, as miR-29a-3p and miR-29c-3p have the same seed sequence. Only target genes with at least a 7mer binding site and the lowest possible hamming distance between human and mouse 3’UTR and binding sites were selected. The 3’UTR reporter constructs were synthesized and cloned into reporter plasmid pMIR-RNL-TK using SpeI and SacI restriction sites by GeneArt (Life Technologies GmbH). The reporter plasmid pMIR-COL1A2, which was identified by ref. ^43^ as direct target gene of miR-29a-3p, served as positive control. The results of the control experiments are given in Extended Data Fig. 7d. The complete list of all tested 3′UTR sequences, including the respective NM accession number, is given in Supplementary Table 9.

### High-throughput miRNA interaction reporter assay

High-throughput analysis of reporter constructs was conducted by a liquid handling system and described previously in ref. ^44^. In brief, HEK 293T cells were seeded at 3.2 × 10^4^ cells per well in a 96-well plate using a liquid handling system epMotion 5,075 (Eppendorf). Twenty-four hours after seeding, cells were transfected with 50 ng per well of either reporter plasmid pMIR-RNL-TK, with or without insert, and 200 ng per well of miRNA expression plasmid pSG5-miR-29a or the empty expression vector pSG5. Forty-eight hours post-transfection, HEK 293T cells were lysed and the lysates were measured using a GloMax Navigator microplate luminometer (Promega) using Luciferase substrates of the Dual-Luciferase Reporter Assay System (Promega). High-throughput miRNA interaction reporter assay was conducted four times in technical duplicates.

### Reporting summary

Further information on research design is available in the Nature Portfolio Reporting Summary linked to this article.

## Online content

Any methods, additional references, Nature Portfolio reporting summaries, source data, extended data, supplementary information, acknowledgements, peer review information; details of author contributions and competing interests; and statements of data and code availability are available at https://doi.org/10.1038/s41587-023-01751-6.

## Supplementary information


Reporting Summary
Supplementary TablesSupplementary Table 1: Metadata for all analyzed samples in the TMS and Parabiosis cohort, with information on tissue, organism, strain, age, sex and cohort. Table 2: Covered sequence length analysis with information for all ncRNAs detected in the TMS cohort; calculated information: Covered read length, reference read length, longest covered region, covered percentage reference length, longest mapping read, total reads mapping and average covered read length. Table 3: Spearman correlation of common miRNAs expressed in all tissues with age, displayed in Fig. 2a. Table 4: Foldchanges and *P* values of parametric two-sided *t*-test and nonparametric two-sided Wilcoxon–Mann–Whitney test for all comparisons 3 months versus all later timepoints in all individual tissues (for all comparisons: sample size per tissue per timepoint *n* > 3). Table 5: miRNA–mRNA interaction pairs identified in all tissues via negative correlation (*r* < -0.5, *P* < 0.05) for global aging miRNAs positively correlated with age (miR-29a-3p, miR-155-5p, miR-184-3p, miR-1895 and miR-29c-3p). Table 6: miRNA–mRNA interaction pairs identified in all tissues via negative correlation (*r* < -0.5, *P* < 0.05) for global aging miRNAs negatively correlated with age (miR-541-3p, miR-487b-3p and miR-300-3p). Table 7: List of paralog identifiers retained for analysis and their alternative paralog identifiers not further considered in the analysis. Table 8: Spearman rank correlation values for miRNAs expressed in each tissue with *P* value (Spearman’s statistics, two-sided). Table 9: List of tested 3′UTR sequences including the respective NM accession number.


## Data Availability

All data generated in this study are freely accessible from the Gene Expression Omnibus (GSE217458, GSE222857). Databases used in this study are miRBase 22 (https://www.mirbase.org/), GtRNSdb 18.1 (http://gtrnadb.ucsc.edu/), RNACentral 15 (https://rnacentral.org/) and Ensembl 100 (https://useast.ensembl.org/index.html).

## Code availability

All analyses have been carried out using freely available software packages. Custom code used to analyze the RNA-seq data and datasets generated and/or processed in the current study is available from the corresponding authors upon request.
