# Supplementary material for: Characterizing expression changes in noncoding RNAs during aging and heterochronic parabiosis across mouse tissues
Source: Nat Biotechnol. 2023 Apr 27;42(1):109–18. doi: 10.1038/s41587-023-01751-6 (PMC10791587; doi:10.1038/s41587-023-01751-6)
Supplement: Supplementary file 1 — Reporting Summary [file 41587_2023_1751_MOESM1_ESM.pdf]

Corresponding author(s): Andreas Keller
Last updated by author(s): Mar 6, 2023

Reporting Summary

Nature Portfolio wishes to improve the reproducibility of the work that we publish. This form provides structure for consistency and transparency in reporting. For further information on Nature Portfolio policies, see our Editorial Policies and the Editorial Policy Checklist.

Statistics

For all statistical analyses, confirm that the following items are present in the figure legend, table legend, main text, or Methods section.

n/a	Confirmed	
☐	☒	The exact sample size (*n*) for each experimental group/condition, given as a discrete number and unit of measurement
☐	☒	A statement on whether measurements were taken from distinct samples or whether the same sample was measured repeatedly
☐	☒	The statistical test(s) used AND whether they are one- or two-sided<br>*Only common tests should be described solely by name; describe more complex techniques in the Methods section.*
☐	☒	A description of all covariates tested
☐	☒	A description of any assumptions or corrections, such as tests of normality and adjustment for multiple comparisons
☐	☒	A full description of the statistical parameters including central tendency (e.g. means) or other basic estimates (e.g. regression coefficient) AND variation (e.g. standard deviation) or associated estimates of uncertainty (e.g. confidence intervals)
☐	☒	For null hypothesis testing, the test statistic (e.g. F, t, r) with confidence intervals, effect sizes, degrees of freedom and P value noted<br>*Give P values as exact values whenever suitable.*
☒	☐	For Bayesian analysis, information on the choice of priors and Markov chain Monte Carlo settings
☒	☐	For hierarchical and complex designs, identification of the appropriate level for tests and full reporting of outcomes
☐	☒	Estimates of effect sizes (e.g. Cohen's *d*, Pearson's *r*), indicating how they were calculated

Our web collection on statistics for biologists contains articles on many of the points above.

Software and code

Policy information about availability of computer code

Data collection	The main data is based on non-coding RNA sequencing results obtained from remaining RNA of the Tabula Muris Senis Study (doi: 10.1038/s41586-020-2496-1) and non-coding RNA sequencing of Parabiosis mice (doi: 10.1038/s41586-022-04461-2). Matching gene expression data was downloaded from previous publications and vesicle data was obtained from another study available as a preprint (doi: 10.1101/2021.05.07.443093). Samples were single-end sequenced on the BGISEQ500RS using the High-throughput Sequencing Set (SE50) (Small RNA) as a service provided by BGI, Hong Kong.
Data analysis	The sequencing data were processed with miRMaster 2.0 in standard settings and mapped read percentages were generated. Data analysis was performed using RStudio Software v4.0.3 with the following packages: viper v1.26.0, data.table v1.14.2, ggrepel v0.9.1, ggvenn v0.1.9, M3C v1.14.0, ggridges v0.5.3, forcats v0.5.1, purrr v0.3.4, tidyr v1.2.0, tibble v3.1.6, ggplot2 v3.3.5, tidyverse v1.3.1, viridisLite v0.4.0, ColorBrewer v1.1-2, reshape2 v1.4.4, pheatmap v1.0.12, Mfuzz v2.52.0 (fuzzy c-means algorithm), DynDoc v1.70.0, widgetTools v1.70.0, e1071 v1.7-9, stringr v1.4.0, dplyr v1.0.8, readr v2.1.2, Biobase v2.52.0. Samples were excluded if fewer than 2 million aligned reads were detected while allowing 1 mismatch per read. Using Bowtie (1.2.3.) reads were mapped against the RNA sequence derived from the respective databases (miRNAs: miRBase 22, tRNAs: GtRNAdb 18.1, piRNA: RNACentral 15, all other ncRNAs: Ensembl 100).

For manuscripts utilizing custom algorithms or software that are central to the research but not yet described in published literature, software must be made available to editors and reviewers. We strongly encourage code deposition in a community repository (e.g. GitHub). See the Nature Portfolio guidelines for submitting code & software for further information.

Data

Policy information about availability of data

All manuscripts must include a data availability statement. This statement should provide the following information, where applicable:

- Accession codes, unique identifiers, or web links for publicly available datasets
- A description of any restrictions on data availability
- For clinical datasets or third party data, please ensure that the statement adheres to our policy

Databases used in this study: miRBase 22 (https://www.mirbase.org/), GtRNSdb 18.1 (http://gtrnadb.ucsc.edu/), RNACentral 15 (https://rnacentral.org/) and Ensembl 100 (https://useast.ensembl.org/index.html).
New Datasets generated in this study are available at GEO: GSE217458, GSE222857.

Human research participants

Policy information about studies involving human research participants and Sex and Gender in Research.

Reporting on sex and gender	N/A
Population characteristics	N/A
Recruitment	N/A
Ethics oversight	N/A

Note that full information on the approval of the study protocol must also be provided in the manuscript.

Field-specific reporting

Please select the one below that is the best fit for your research. If you are not sure, read the appropriate sections before making your selection.

☒ Life sciences ☐ Behavioural & social sciences ☐ Ecological, evolutionary & environmental sciences

For a reference copy of the document with all sections, see nature.com/documents/nr-reporting-summary-flat.pdf

Life sciences study design

All studies must disclose on these points even when the disclosure is negative.

Sample size	Sample size was used as described in previous publication (Schaum, N., Lehallier, B., Hahn, O. et al. Ageing hallmarks exhibit organ-specific temporal signatures. Nature 583, 596–602 (2020).). No statistical methods were used to pre-determine sample size. The number of mice was restricted due to costs and practical constraints of collecting and processing organs. However, the study is designed such that there are many closely related experimental groups (up to 10 different ages in close proximity across the lifespan), allowing for pooling of closely related ages, and for analyzes across the lifespan that include >50 samples.
Data exclusions	Samples with less than two million reads were excluded.
Replication	Up to six mice per time-point were processed as biological replicates, information on all replicates is provided in Supplementary Table 1.
Randomization	Randomization was performed in the case of mouse dissection order and during the preparation of 96-well plates for cDNA creation.
Blinding	Experimenter was blinded to samples during RNA processing and library generation. Blinding was not applicable for the transcriptomic data and non-coding RNA analysis.

Behavioural & social sciences study design

All studies must disclose on these points even when the disclosure is negative.

Study description	*Briefly describe the study type including whether data are quantitative, qualitative, or mixed-methods (e.g. qualitative cross-sectional, quantitative experimental, mixed-methods case study).*
Research sample	*State the research sample (e.g. Harvard university undergraduates, villagers in rural India) and provide relevant demographic*

Research sample	*information (e.g. age, sex) and indicate whether the sample is representative. Provide a rationale for the study sample chosen. For studies involving existing datasets, please describe the dataset and source.*
Sampling strategy	*Describe the sampling procedure (e.g. random, snowball, stratified, convenience). Describe the statistical methods that were used to predetermine sample size OR if no sample-size calculation was performed, describe how sample sizes were chosen and provide a rationale for why these sample sizes are sufficient. For qualitative data, please indicate whether data saturation was considered, and what criteria were used to decide that no further sampling was needed.*
Data collection	*Provide details about the data collection procedure, including the instruments or devices used to record the data (e.g. pen and paper, computer, eye tracker, video or audio equipment) whether anyone was present besides the participant(s) and the researcher, and whether the researcher was blind to experimental condition and/or the study hypothesis during data collection.*
Timing	*Indicate the start and stop dates of data collection. If there is a gap between collection periods, state the dates for each sample cohort.*
Data exclusions	*If no data were excluded from the analyses, state so OR if data were excluded, provide the exact number of exclusions and the rationale behind them, indicating whether exclusion criteria were pre-established.*
Non-participation	*State how many participants dropped out/declined participation and the reason(s) given OR provide response rate OR state that no participants dropped out/declined participation.*
Randomization	*If participants were not allocated into experimental groups, state so OR describe how participants were allocated to groups, and if allocation was not random, describe how covariates were controlled.*

Ecological, evolutionary & environmental sciences study design

All studies must disclose on these points even when the disclosure is negative.

Study description	*Briefly describe the study. For quantitative data include treatment factors and interactions, design structure (e.g. factorial, nested, hierarchical), nature and number of experimental units and replicates.*
Research sample	*Describe the research sample (e.g. a group of tagged Passer domesticus, all Stenocereus thurberi within Organ Pipe Cactus National Monument), and provide a rationale for the sample choice. When relevant, describe the organism taxa, source, sex, age range and any manipulations. State what population the sample is meant to represent when applicable. For studies involving existing datasets, describe the data and its source.*
Sampling strategy	*Note the sampling procedure. Describe the statistical methods that were used to predetermine sample size OR if no sample-size calculation was performed, describe how sample sizes were chosen and provide a rationale for why these sample sizes are sufficient.*
Data collection	*Describe the data collection procedure, including who recorded the data and how.*
Timing and spatial scale	*Indicate the start and stop dates of data collection, noting the frequency and periodicity of sampling and providing a rationale for these choices. If there is a gap between collection periods, state the dates for each sample cohort. Specify the spatial scale from which the data are taken*
Data exclusions	*If no data were excluded from the analyses, state so OR if data were excluded, describe the exclusions and the rationale behind them, indicating whether exclusion criteria were pre-established.*
Reproducibility	*Describe the measures taken to verify the reproducibility of experimental findings. For each experiment, note whether any attempts to repeat the experiment failed OR state that all attempts to repeat the experiment were successful.*
Randomization	*Describe how samples/organisms/participants were allocated into groups. If allocation was not random, describe how covariates were controlled. If this is not relevant to your study, explain why.*
Blinding	*Describe the extent of blinding used during data acquisition and analysis. If blinding was not possible, describe why OR explain why blinding was not relevant to your study.*

Did the study involve field work? ☐ Yes ☐ No

Field work, collection and transport

Field conditions	*Describe the study conditions for field work, providing relevant parameters (e.g. temperature, rainfall).*
Location	*State the location of the sampling or experiment, providing relevant parameters (e.g. latitude and longitude, elevation, water depth).*
Access & import/export	*Describe the efforts you have made to access habitats and to collect and import/export your samples in a responsible manner and in*

Access & import/export	*compliance with local, national and international laws, noting any permits that were obtained (give the name of the issuing authority, the date of issue, and any identifying information).*
Disturbance	*Describe any disturbance caused by the study and how it was minimized.*

Reporting for specific materials, systems and methods

We require information from authors about some types of materials, experimental systems and methods used in many studies. Here, indicate whether each material, system or method listed is relevant to your study. If you are not sure if a list item applies to your research, read the appropriate section before selecting a response.

Materials & experimental systems

n/a	Involved in the study
☒	☐ Antibodies
☐	☒ Eukaryotic cell lines
☒	☐ Palaeontology and archaeology
☐	☒ Animals and other organisms
☒	☐ Clinical data
☒	☐ Dual use research of concern

Methods

n/a	Involved in the study
☒	☐ ChIP-seq
☒	☐ Flow cytometry
☒	☐ MRI-based neuroimaging

Eukaryotic cell lines

Policy information about cell lines and Sex and Gender in Research

Cell line source(s)	HEK 293T (DSMZ - German Collection of Microorganisms and Cell Cultures GmbH)
Authentication	The human HEK 293T cell lines were purchased from the German collection of microorganisms and cell cultures (DSMZ). The authentication of these cell lines was ensured using STR DNA typing by the DSMZ.
Mycoplasma contamination	Tested negative.
Commonly misidentified lines (See ICLAC register)	No commonly misidentified cell lines were used in this study.

Animals and other research organisms

Policy information about studies involving animals; ARRIVE guidelines recommended for reporting animal research, and Sex and Gender in Research

Laboratory animals	Aging cohort: C57BL/6JN males (n=4, aged 1, 3, 6, 9, 12, 15, 18, 21, 24, 27 months; and females (n=2, ages 1, 3, 6, 9, 12, 15, 18, 21 months)<br>Parabiosis cohort: C57BL/6JN males (aged 3 months (n=4), 4.5 months (n=4), 19 months (n=12))<br>C57BL/6-Tg(UBC-GFP)30Scha/J males (aged 4 months (n=7))<br>C57BL/6J males (aged 19 months (n=3))<br>Circulating miRNA study: C57BL/6N females (aged 2 months (n=3), aged 6 months (n=4), aged 8 months (n=3), aged 12 months (n=2), aged 18 months (n=2))
Wild animals	None
Reporting on sex	All sample information is available in Supplementary Table 1.
Field-collected samples	None
Ethics oversight	VA Palo Alto Committee on Animal Research

Note that full information on the approval of the study protocol must also be provided in the manuscript.
